# Transplantation of Elderly Human Gut Microbiota Into Pigs Reprograms Intestinal Barrier Function, Plasma Metabolome, and Gut Mucosal Transcriptomic Landscape

**DOI:** 10.1111/acel.70725

**Published:** 2026-09-23

**Authors:** Jiaxin Wang, Lingyu Li, Lihua Mei, Zhining Tang, Baorui Liao, Qianye Zhao, Yuzeng Wang, Qingyao Fu, Liu Ren, Zhian Zhai, Yifei Xu, Aoyu Yang, Shuai Duan, Zhengyuan Zhai, Yanling Hao, Yalin Zhou, Yajun Xu, Ying Yang, Zhenlong Wu, Yun Ji

**Affiliations:** ^1^ State Key Laboratory of Animal Nutrition and Feeding China Agricultural University Beijing China; ^2^ College of Food Science and Nutritional Engineering China Agricultural University Beijing China; ^3^ Key Laboratory of Precision Nutrition and Food Quality, Department of Nutrition and Health China Agricultural University Beijing China; ^4^ Department of Nutrition and Food Hygiene, School of Public Health Peking University Beijing China

**Keywords:** aging, fecal microbiota transplantation, gut microbiota, intestinal barrier, pig model

## Abstract

The gut microbiota plays a pivotal role in maintaining intestinal homeostasis and regulating host metabolism, yet its composition and function undergo substantial alterations with aging. However, the relationship between age‐associated microbial changes and host intestinal physiology remains not fully elucidated. Here, we employed Bama miniature pigs, a model with close gastrointestinal similarity to humans, to investigate the impact of fecal microbiota transplantation (FMT) from young or elderly human donors on gut structure, barrier integrity, plasma metabolites, and intestinal mucosal transcriptomics. FMT resulted in distinct gut microbial profiles, with elderly‐donor FMT reducing ileal villus height and tight junction proteins (ZO‐1, claudin‐1, and occludin) across multiple intestinal segments. At the species level, *Phocea massiliensis* predominated in young‐donor pigs, while *Blautia obeum* was enriched in elderly‐donor pigs. Plasma metabolomic analysis revealed increased 3‐methyloxindole, prostaglandin E3, and 2‐hydroxybutanoic acid but reduced Tyr‐Phe and 2‐hydroxyoctadecanoic acid in the elderly group. Among *B. obeum*‐associated metabolites, Tyr‐Phe and 2‐hydroxyoctadecanoic acid exhibited a positive correlation with certain intestinal tight junction protein levels, whereas prostaglandin E3 showed an inverse correlation. Further validation performed in IPEC‐1 cells demonstrated that Tyr‐Phe elevated transepithelial electrical resistance (TEER), while prostaglandin E3 lowered this parameter. Transcriptomic profiling identified seven hub genes (*MX2*, *ISG15*, *IFI6*, *IFIT1*, *OAS1*, *DHX58*, *ISG12(A)*) that were consistently downregulated in the ileal mucosa of pigs receiving elderly‐donor microbiota. These findings link age‐associated microbiota alterations to coordinated changes in intestinal architecture, barrier integrity, and host metabolic and transcriptional profiles, providing insights into microbial and metabolic features associated with age‐related intestinal decline.

## Graphical abstract

Elderly human donor microbiota disrupts intestinal barrier integrity and reshapes host metabolic and mucosal transcriptional profiles in human microbiota‐associated pigs. Integrated multi‐omics analyses identify coordinated microbial, metabolic, and host alterations associated with age‐related intestinal decline.

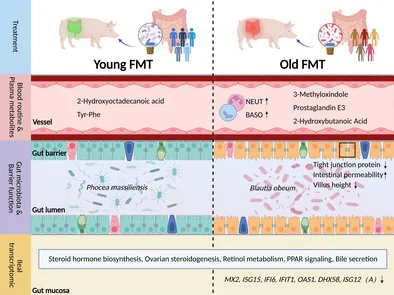

## 1 Introduction

With the rapid progression of global population aging, the number of elderly individuals is steadily increasing. Maintaining intestinal health is a cornerstone of overall well‐being in the elderly. Age‐related decline in intestinal function is closely associated with chronic enteritis (Zheng et al. 2022), impaired nutrient absorption (Cristina and Lucia 2021), and metabolic disorders (Li et al. 2025), which collectively compromise quality of life and may shorten life expectancy. Thus, elucidating the physiological and pathological changes of the aging intestine and identifying the determinants of intestinal health are essential for designing effective dietary and medical interventions.

The intestinal barrier serves as the first line of defense against luminal insults, preventing pathogen invasion and maintaining intestinal homeostasis (Elshaer and Begun 2017). However, aging is accompanied by progressive deterioration of barrier integrity, which results from gut microbiota dysbiosis, immune senescence, and disrupted cellular metabolism (Khaledi et al. 2024). This impairment is closely linked with systemic diseases in the elderly, including diabetes and cardiovascular disorders (Zhang et al. 2023). Increased intestinal permeability facilitates the translocation of bacteria and endotoxins such as lipopolysaccharide into the bloodstream, which induces chronic low‐grade inflammation (Martel et al. 2022). This inflammatory response contributes to the deterioration of metabolic function and immune dysregulation, thereby accelerating the development and progression of age‐related diseases.

The gut microbiota is recognized as a pivotal factor in regulating host physiology, including metabolism, immune responses, and barrier function (Alam and Neish 2018; Ghosh et al. 2022; Nicolas and Chang 2019; Wu and Wu 2012). Aging induces profound alterations in microbial diversity and composition (Li et al. 2021; Odamaki et al. 2016). These alterations are linked to compromised intestinal barrier function, systemic inflammatory responses, and metabolic dysregulation (Juarez‐Fernandez et al. 2021; Thevaranjan et al. 2017). Although these associations are increasingly recognized, the causal impact of aging‐associated microbiota on intestinal structure, barrier function, and host metabolism remains insufficiently understood.

A major challenge stems from the absence of direct evidence illustrating the influence of age‐specific gut microbiota on host intestinal physiology. Fecal microbiota transplantation (FMT), which transfers intact donor microbial communities to recipients (Borody and Khoruts 2011), provides a unique opportunity to reconstruct age‐associated gut ecosystems in vivo while preserving microbial complexity and functional capacity. Pigs represent an optimal biomedical model for studying human gastrointestinal physiology owing to their remarkable anatomical, cellular, and molecular similarities with humans. These include comparable small intestine length‐to‐body weight ratios, the presence of well‐developed submucosal glands and villus structures, and similar cell populations, as well as a high degree of genomic homology (Gonzalez et al. 2015; Walters et al. 2012). Building upon these similarities, the transplantation of fecal microbiota from humans of different age groups into porcine models provides a distinctive and controlled methodology to systematically assess how age‐associated microbial communities affect intestinal morphology, barrier integrity, mucosal gene expression, and host metabolic profiles. Therefore, the present study aimed to investigate the effects of fecal microbiota from young and elderly human donors on the intestinal structure and barrier function of pigs. By integrating microbiome, metabolome, and transcriptome analyses, this study sought to delineate the interactions between donor age‐related microbiota and host intestinal physiology. These findings are expected to provide new mechanistic insights into the role of aging‐associated microbiota in intestinal health and to offer theoretical support for the development of targeted nutritional and therapeutic strategies for the elderly.

## 2 Results

### 2.1 Impact of Young‐ Versus Elderly‐Derived Fecal Microbiota Transplantation on Hematological and Biochemical Profiles in Pigs

As shown in Table S1, most hematological parameters were comparable among the non‐FMT control pigs and pigs receiving FMT from young or elderly donors. Notably, the elderly donor group exhibited significantly higher absolute neutrophil and basophil counts than the young donor group (*p* < 0.05). No significant differences were observed in erythrocyte‐related indices, total leukocyte count, leukocyte percentages, lymphocyte, monocyte, or eosinophil counts, or platelet‐associated parameters. Similarly, plasma biochemical parameters related to hepatic function, renal function, lipid metabolism, glucose metabolism, and protein metabolism remained unchanged among the three groups (Table S2). These results indicate that FMT from elderly donors selectively affected specific circulating innate immune cell populations, without inducing broad hematological or systemic biochemical alterations in recipient pigs.

### 2.2 Alterations in Gut Microbial α‐ and β‐Diversity Following Antibiotics and Human‐Derived Fecal Microbiota Transplantation in Pigs

**FIGURE 1:**
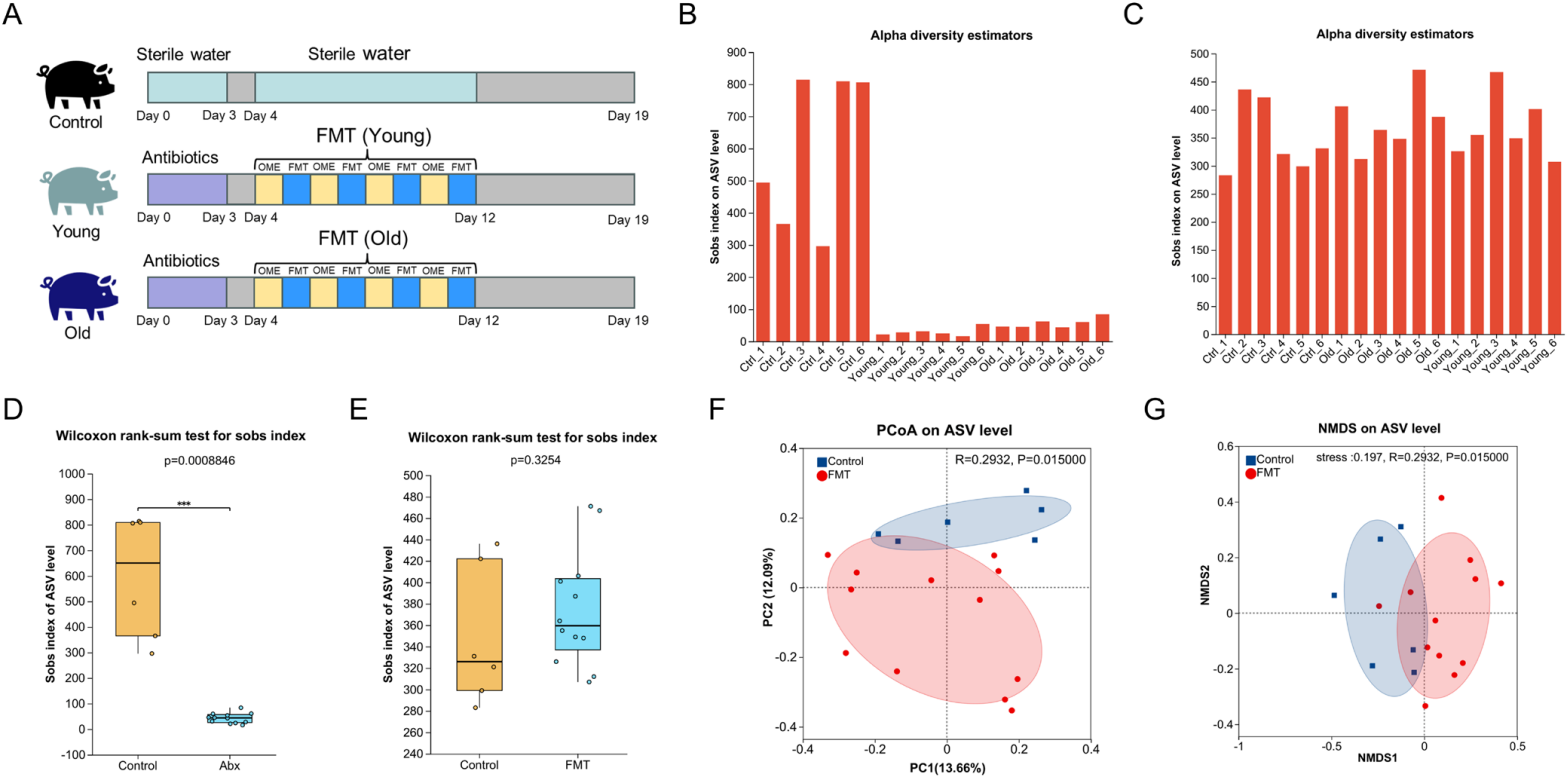
Experimental design and effects of antibiotic treatment and fecal microbiota transplantation (FMT) on gut microbial diversity in pigs. (A) Schematic overview of the experimental workflow. (B, D) Changes in Sobs index before and after antibiotic administration. Abx, antibiotic‐treated pigs; Control, pigs prior to antibiotic exposure. (C, E) Sobs index of pigs receiving human FMT (young or elderly donors) compared with untreated controls. (F, G) Principal coordinates analysis (PCoA) and non‐metric multidimensional scaling (NMDS), both based on Bray–Curtis dissimilarities, depicting differences in microbial community structure among groups; statistical significance was evaluated using analysis of similarities (ANOSIM) (*n* = 6). ***p < 0.001.

We next evaluated the impact of antibiotic treatment and FMT on the diversity of the porcine gut microbiota. Following antibiotic administration, a marked reduction in microbial diversity was observed, with the Sobs index significantly decreased in the antibiotic‐treated group compared with controls (*p <* 0.001; Figure 1B,D). Consistently, ACE‐based rarefaction analysis further demonstrated a pronounced reduction in microbial richness after antibiotics treatment, with the ACE richness curves of the antibiotics‐treated groups markedly lower than those of the control group and remaining at extremely low levels (Figure S1A). One week after FMT, no significant difference in the Sobs index was detected between the FMT group and controls (Figure 1C,E), suggesting that α‐diversity of the gut microbiota was restored to baseline levels. Similarly, ACE‐based rarefaction curves showed comparable richness profiles between FMT recipients and control animals (Figure S1B), further supporting the restoration of microbial richness following transplantation. By contrast, β‐diversity analysis revealed distinct community structures. Principal coordinate analysis (PCoA) and non‐metric multidimensional scaling (NMDS) based on ASV profiles demonstrated clear separation between pigs receiving human‐derived FMT and controls (*p <* 0.05; Figure 1F,G), indicating that while overall microbial richness was restored, the compositional structure of the gut microbiota was substantially reshaped by the transplanted human microbiota.

### 2.3 Remodeling of Pig Intestinal Microbiota by Fecal Microbiota Transplantation From Human Donors

**FIGURE 2:**
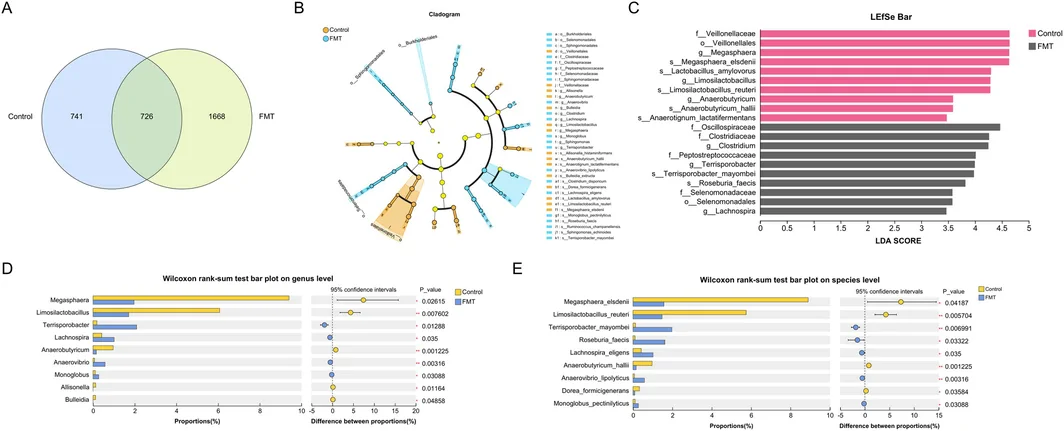
Alterations in gut microbial composition following human fecal microbiota transplantation. (A) Venn diagram of ASVs in FMT‐treated and control pigs. (B) Cladogram depicting phylogenetic distribution of taxa differing between groups. (C) Linear discriminant analysis effect size (LEfSe) was applied to identify discriminative taxa, using an all‐against‐all strategy for multiclass comparisons. (D, E) Differentially abundant genera and species were determined by Wilcoxon rank‐sum tests, with *p* values adjusted using the false discovery rate (FDR) method (*n* = 6). *p < 0.05; **p < 0.01.

A total of 3135 amplicon sequence variants (ASVs) were identified across all samples. The overlap and distinctness of microbial communities are illustrated by the Venn diagram (Figure 2A). Specifically, the control group contained 1467 ASVs, whereas the FMT group harbored 2394 ASVs. Among these, 741 ASVs were unique to the control group, accounting for 50.51% of its total ASVs, while 1668 ASVs were unique to the FMT group, representing 69.67% of its total ASVs. Only 726 ASVs were shared between the groups (corresponding to 23.16% of all identified taxa), indicating substantial divergence in community membership following FMT (Figure 2A). Consistent with this overall shift, LEfSe analysis and the phylogenetic cladogram identified distinct microbial signatures associated with human‐derived FMT (Figure 2B,C). FMT‐treated pigs were characterized by enrichment of several taxa, particularly members of Oscillospiraceae, Clostridiaceae, Peptostreptococcaceae, Selenomonadaceae, and the genera *Terrisporobacter* and *Lachnospira*. At the species level, *Terrisporobacter mayombei* and *Roseburia faecis* were among the representative taxa enriched in the FMT group. In contrast, control pigs showed preferential enrichment of Veillonellaceae‐related taxa, including *Megasphaera* and *Megasphaera elsdenii* , as well as *Limosilactobacillus reuteri*, *Anaerobutyricum hallii*, and *Anaerotignum lactatifermentans*. Wilcoxon rank‐sum analysis further corroborated these FMT‐associated compositional changes (Figure 2D,E). Specifically, FMT significantly increased the relative abundance of *Terrisporobacter*, *Lachnospira*, *Anaerovibrio*, and *Monoglobus*, together with *T. mayombei*, *R. faecis* , *Lachnospira eligens*, *Anaerovibrio lipolyticus* , and *Monoglobus pectinilyticus* (*p* < 0.05 or *p* < 0.01). Conversely, *Megasphaera*, *Limosilactobacillus*, and *Anaerobutyricum*, including *M. elsdenii* , *L. reuteri* , and *A. hallii* , were significantly depleted after FMT (*p* < 0.05 or *p* < 0.01). Collectively, these findings demonstrate that human‐derived FMT substantially reconfigured both the membership and taxonomic structure of the porcine gut microbiota, yielding a distinct microbial profile characterized by enrichment of specific FMT‐associated core taxa.

### 2.4 Age‐Dependent Effects of Donor Fecal Microbiota on Gut Microbial Diversity and Community Health in Pigs

**FIGURE 3:**
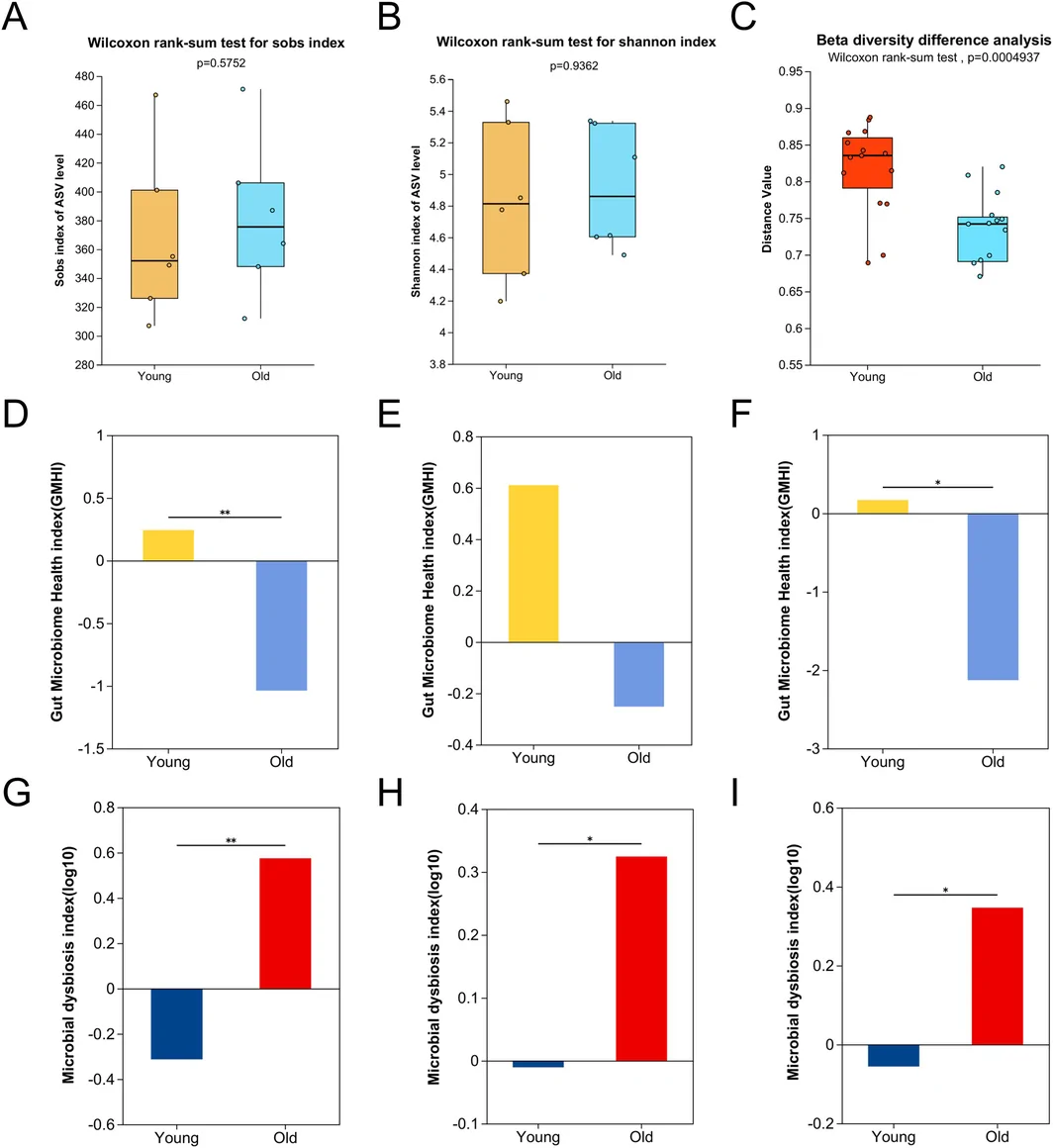
Effects of young‐ and elderly‐donor fecal microbiota transplantation on intestinal microbiota diversity and health indices. (A, B) α‐diversity measured by Sobs and Shannon indices. (C) β‐Diversity analysis based on Bray–Curtis dissimilarities, illustrating differences in microbial community structure among groups. (D–F) Gut microbiome health index (GMHI) at ASV, genus, and species levels. (G–I) Microbiota dysbiosis index (MDI) at corresponding taxonomic levels (*n* = 6). *p < 0.05; **p < 0.01.

Analysis of α‐diversity revealed no significant differences in either the Sobs index (Figure 3A) or the Shannon index (Figure 3B) between pigs transplanted with fecal microbiota from young and elderly donors, indicating comparable overall richness and evenness of gut microbial communities. In contrast, β‐diversity analysis (Figure 3C) demonstrated that microbial structural diversity was significantly lower in the elderly donor group than in the young donor group (*p <* 0.05), suggesting that donor age may affect the compositional stability of the recipient gut microbiota. With respect to microbial health indices, pigs receiving fecal transplants from elderly donors exhibited significantly reduced gut microbiota health index (GMHI) values at both the ASV and species levels (Figure 3D,F; *p <* 0.05), compared with pigs receiving microbiota from young donors. Conversely, the microbial dysbiosis index (MDI) was markedly elevated in the elderly donor group at the ASV, genus, and species levels (Figure 3G–I; *p <* 0.05). However, as GMHI and MDI were originally established from human microbiome datasets and have not been validated in cross‐species FMT models, these indices were interpreted as exploratory measures of microbiota‐associated health status in the present human‐to‐pig FMT model.

**FIGURE 4:**
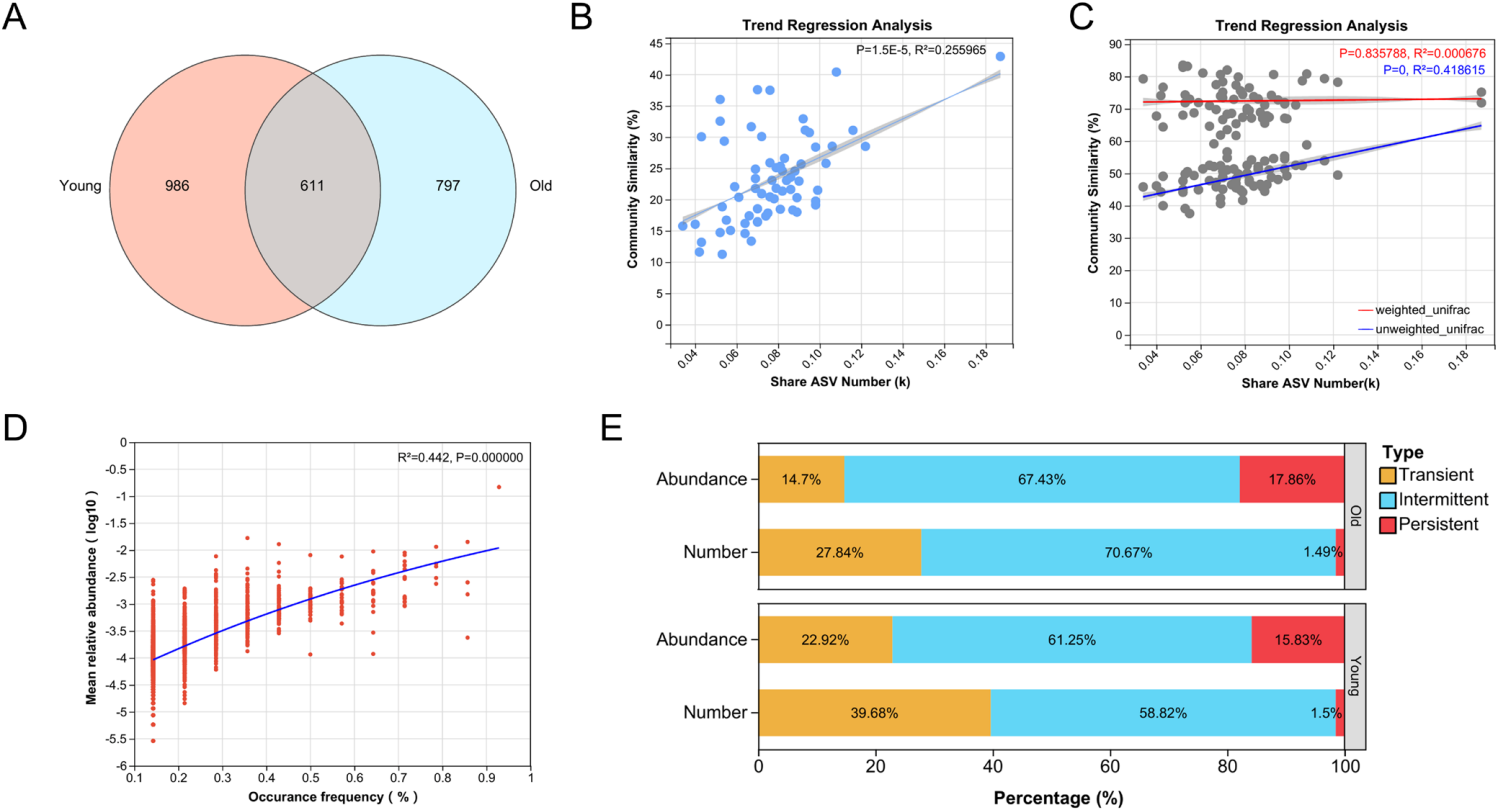
Community composition, similarity, and prevalence of gut microbiota after young‐ or elderly‐donor FMT. (A) Venn diagram showing distinct and shared ASVs between donor groups. (B) Bray–Curtis analysis linking the number of shared ASVs to community similarity. (C) Weighted UniFrac revealing conserved abundance‐weighted phylogeny, while unweighted UniFrac highlights differences in taxonomic presence and phylogenetic distribution. (D) Relationship between ASV occurrence frequency and mean relative abundance across all samples. (E) Classification of ASVs into transient, intermittent, and persistent types with group comparisons of their proportions by abundance and number (*n* = 6).

Venn diagram analysis (Figure 4A) further showed that 986 amplicon sequence variants (ASVs) were unique to the young donor group, while 797 ASVs were specific to the elderly donor group, with 611 ASVs shared between the two groups. These findings indicate that fecal microbiota from different age groups shape distinct core microbial communities in the host. We analyzed the community similarity trends of gut microbiota following transplantation with fecal microbiota from young versus elderly donors. Based on Bray–Curtis distance, a significant positive correlation was observed between the number of shared ASVs and community similarity (*p* = 1.5 × 10^−5^, *R* ^2^ = 0.256) (Figure 4B), indicating that increased shared taxa strongly enhanced overall community resemblance. In contrast, weighted UniFrac analysis showed no significant relationship (*p* = 0.835788, *R* ^2^ = 0.000676) (Figure 4C), suggesting the abundance‐weighted phylogenetic structure of dominant taxa remained largely conserved. However, unweighted UniFrac analysis revealed a highly significant positive correlation (*p* = 0, *R* ^2^ = 0.418615) (Figure 4C), highlighting pronounced differences in taxonomic presence/absence and phylogenetic distribution. Collectively, these findings suggest that while dominant taxa abundance patterns remain stable following FMT from donors of different ages, donor age exerts a strong influence on the reconstituted gut microbiota by shaping rare taxa and phylogenetic lineages. Next, we explored the relationship between species occurrence frequency and their relative abundance across all samples by performing a regression analysis. A significant positive correlation was observed between occurrence frequency and mean relative abundance (*R* ^2^ = 0.442, *p <* 0.001) (Figure 4D). To assess the environmental sensitivity of the gut microbial communities, we classified the amplicon sequence variants (ASVs) into transient, intermittent, and persistent types based on their prevalence. The relative proportions of these categories in terms of both abundance and number were compared between pigs transplanted with fecal microbiota from young and elderly human donors (Figure 4E). In the elderly donor group, transient ASVs constituted 27.84% of the total ASV number and 14.7% of the abundance, while intermittent ASVs dominated the community (70.67% in number and 67.43% in abundance). Persistent ASVs accounted for only 1.49% of the ASV number but contributed 17.86% of the abundance. In the young donor group, transient ASVs were relatively lower in abundance (22.92%) and showed a slightly higher proportion in number (39.68%). Intermittent ASVs represented 58.82% of the ASV number and 61.25% of the abundance. Persistent ASVs, though only 1.5% of the ASV number, contributed 15.83% of the abundance. Overall, the gut microbial communities from young donors harbored a markedly higher proportion of transient ASVs compared with those from elderly donors, both in terms of number and relative abundance. Since a higher proportion of transient ASVs indicates stronger environmental sensitivity, these findings suggest that the microbiota transplanted from young individuals are more environmentally sensitive. By contrast, communities derived from elderly donors contained relatively fewer transient ASVs, implying reduced sensitivity to environmental fluctuations. Notably, the proportion of persistent ASVs was comparable between the two groups, suggesting that the stability of the core microbiota was not substantially different.

### 2.5 Comparation of the Gut Microbiota of Pigs Receiving Young and Elderly Donor Microbiota

**FIGURE 5:**
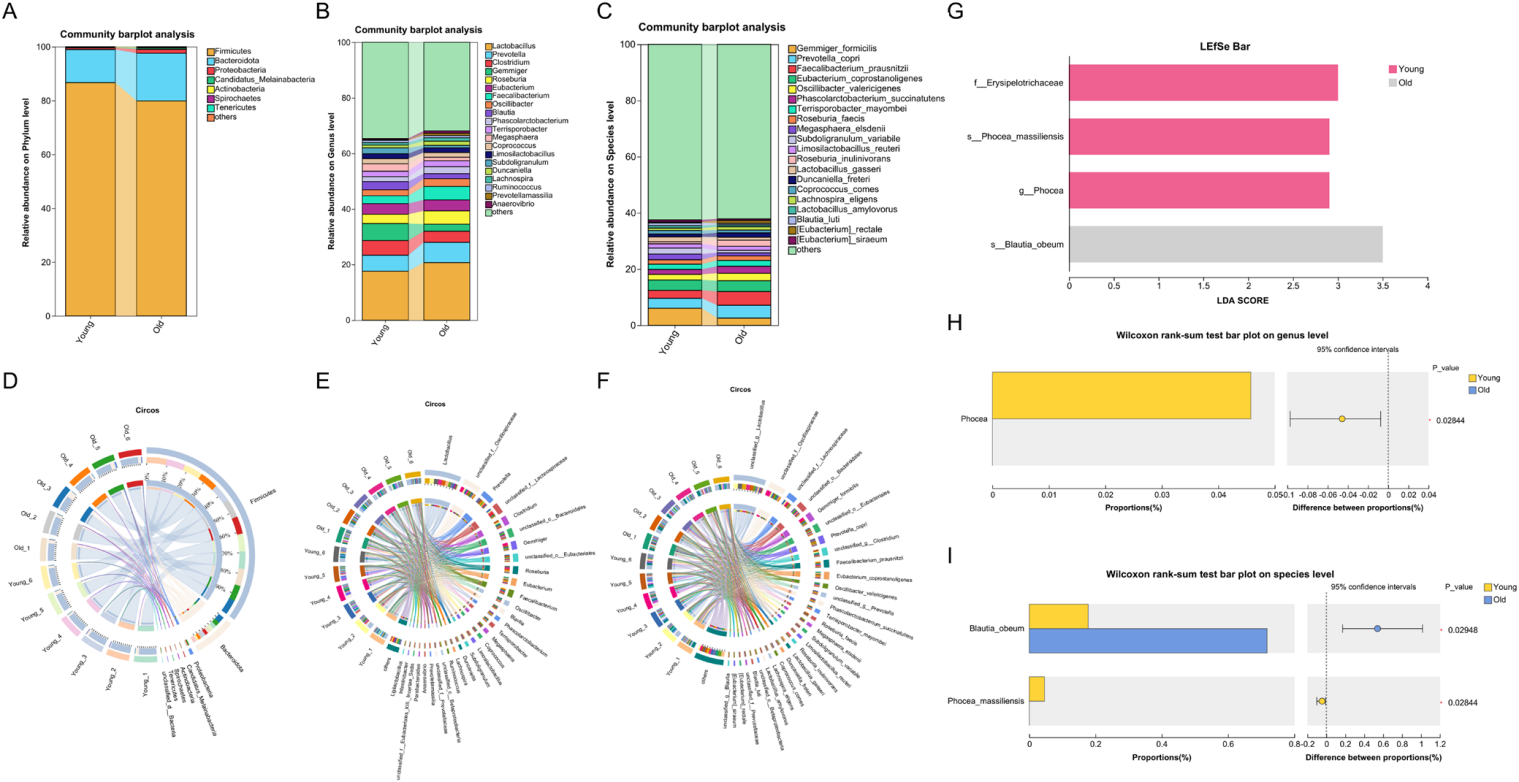
Taxonomic differences in gut microbiota of pigs receiving young‐ or elderly‐donor FMT. (A–C) Bar plots of microbial composition at phylum, genus, and species levels. (D–F) Circos diagrams showing sample‐taxa associations at corresponding taxonomic ranks. (G) Linear discriminant analysis effect size (LEfSe) was employed to delineate taxa displaying differential enrichment, using an all‐against‐all strategy for multiclass comparison. (H, I) Genus‐ and species‐level differences in relative abundance were evaluated using Wilcoxon rank‐sum tests, with adjusted *p* values derived through false discovery rate (FDR) correction (*n* = 6).

Community barplot analysis revealed the average relative abundances of gut microbial communities at the phylum, genus, and species levels in pigs receiving fecal microbiota transplants from either young or aged donors (Figure 5A–C). Circos plots illustrated the taxonomic distribution of gut microbiota at the phylum, genus, and species levels across the two groups (Figure 5D–F). To further identify discriminative microbial taxa, LEfSe analysis was performed. The young donor group was enriched with members of the family Erysipelotrichaceae, as well as *Phocea massiliensis* and the genus *Phocea*. In contrast, the elderly donor group was characterized by the enrichment of *Blautia obeum* (Figure 5G). Wilcoxon rank‐sum testing further validated these findings. At the genus level, the relative abundance of *Phocea* was significantly reduced in pigs receiving fecal microbiota from elderly donors compared with those receiving microbiota from young donors (*p <* 0.05; Figure 5H). At the species level, pigs transplanted with elderly donor microbiota exhibited a significant increase in *B. obeum* abundance (*p <* 0.05), accompanied by a marked reduction in *P. massiliensis* (*p <* 0.05; Figure 5I).

### 2.6 Effect of Young or Elderly Donor Microbiota Transplantation on Intestinal Architecture in Pigs

**FIGURE 6:**
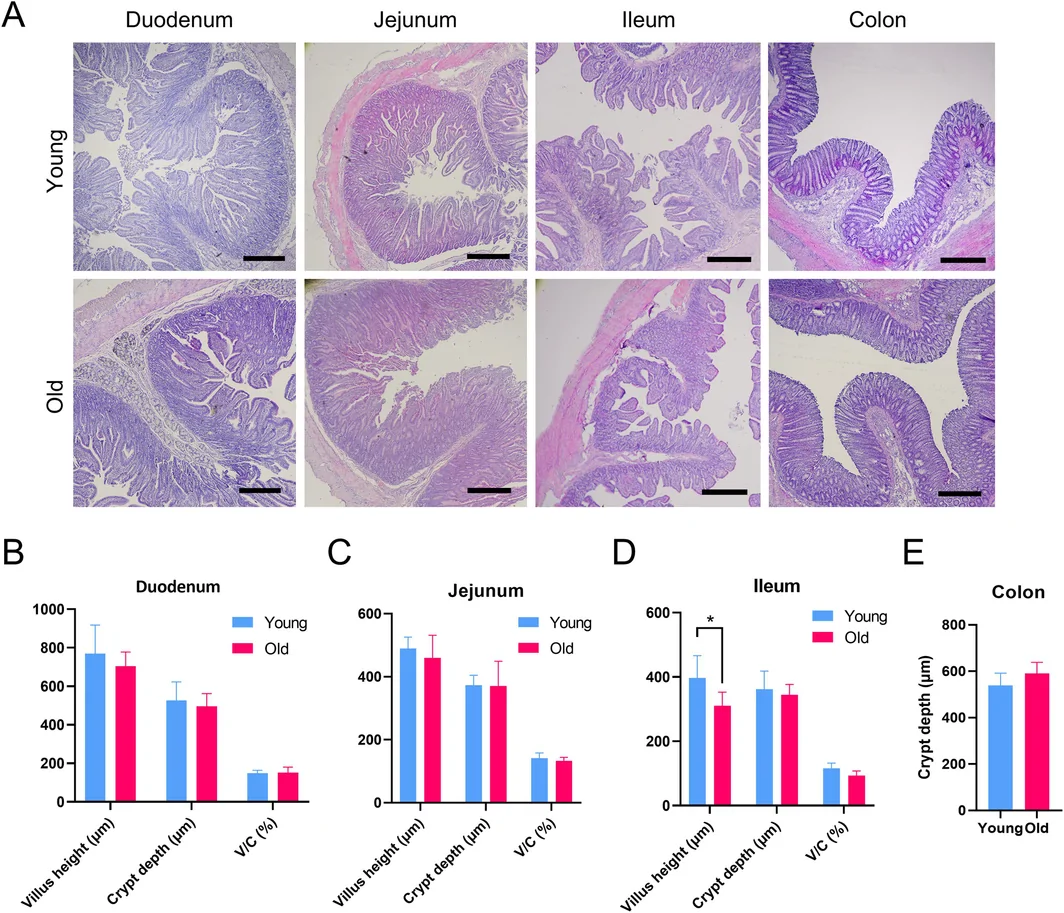
Histological changes in the intestine of pigs following fecal microbiota transplantation. (A) Representative H&E‐stained images of duodenum, jejunum, ileum, and colon (scale bar, 200 μm). (B–D) Quantification of villus height, crypt depth, and villus‐to‐crypt ratios in duodenum, jejunum, and ileum. (E) Crypt depth in the colon (*n* = 6). *p < 0.05.

Histological examination of the small intestine (duodenum, jejunum, and ileum) and colon was performed using H&E staining (Figure 6A). Morphometric analysis revealed no significant differences between pigs receiving young versus elderly donor microbiota in villus height, crypt depth, or the villus height‐to‐crypt depth ratio in the duodenum and jejunum (Figure 6B,C). In contrast, ileal villus height was significantly reduced in pigs transplanted with elderly donor microbiota compared with those receiving young donor microbiota (*p <* 0.05), whereas ileal crypt depth and the villus height‐to‐crypt depth ratio did not differ significantly between groups (Figure 6D). Moreover, colonic crypt depth was comparable between the two groups (Figure 6E). These findings indicate that fecal microbiota from elderly donors may selectively impair ileal villus architecture, while having minimal effects on proximal small intestine or colonic morphology, suggesting a region‐specific impact of donor age on intestinal structure.

### 2.7 Donor Age‐Mediated Modulation of Intestinal Barrier Function in Pigs

**FIGURE 7:**
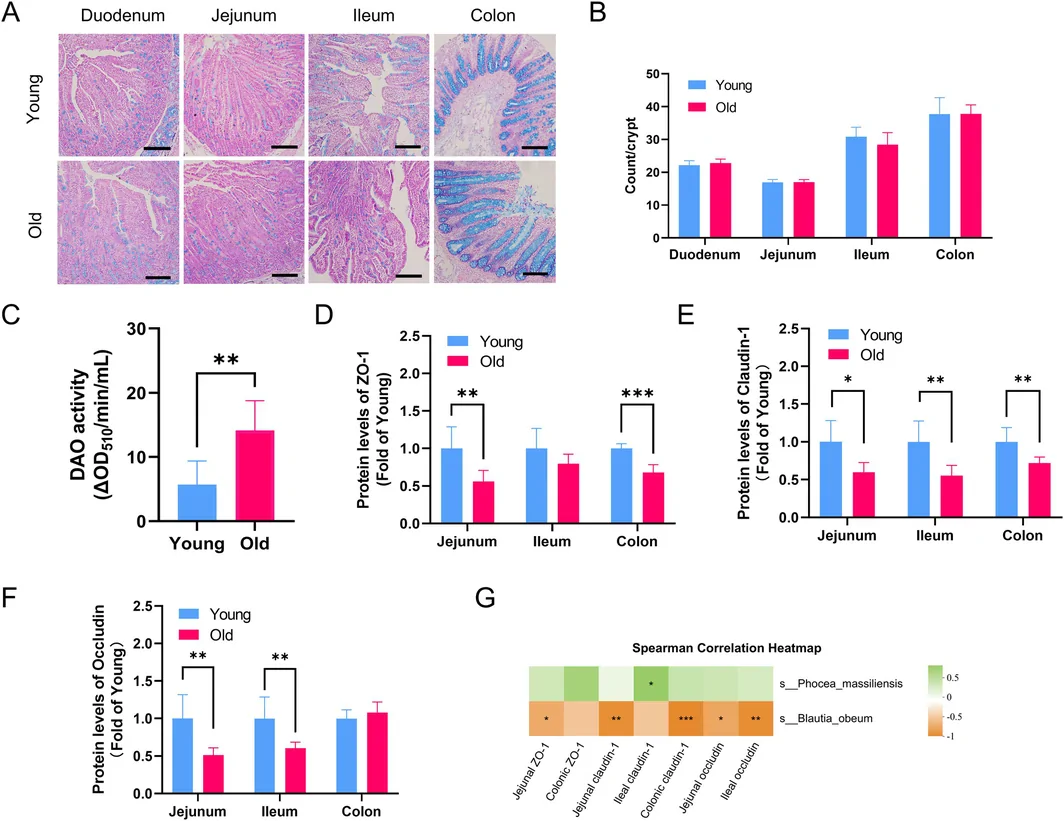
Impact of fecal microbiota transplantation on intestinal barrier function. (A) Representative alcian blue staining of mucin in duodenum, jejunum, ileum, and colon (scale bar, 200 μm). (B) Quantitative analysis of mucin content. *n* = 6. (C) Plasma diamine oxidase (DAO) concentration. *n* = 5–6. (D–F) ELISA quantification of ZO‐1, Claudin‐1, and Occludin in jejunal, ileal, and colonic mucosa. *n* = 6. (G) Heatmap showing Spearman correlations between species‐level differential taxa and tight junction protein expression. *n* = 6. *p < 0.05; **p < 0.01; ***p < 0.001.

Figure 7 illustrates the impact of fecal microbiota from young or elderly donors on intestinal barrier function in pigs. The number of goblet cells in the duodenum, jejunum, ileum, and colon did not differ significantly between the elderly and young donor groups (Figure 7A,B). However, serum diamine oxidase (DAO) levels, an indicator of intestinal mucosal integrity and barrier disruption, were significantly elevated in pigs receiving elderly donor microbiota compared with those receiving young donor microbiota (*p <* 0.01; Figure 7C), suggesting increased intestinal permeability associated with elderly‐derived microbiota. Protein levels of key tight junction markers exhibited segment‐specific alterations. Jejunal tissues from pigs transplanted with elderly donor microbiota displayed significantly reduced levels of ZO‐1 (*p <* 0.01), Claudin‐1 (*p <* 0.05), and Occludin (*p <* 0.01). In the ileum, Claudin‐1 (*p <* 0.01) and Occludin (*p <* 0.01) were markedly decreased compared with the young donor group, whereas colonic ZO‐1 (*p <* 0.001) and Claudin‐1 (*p <* 0.01) were also significantly downregulated (Figure 7D–F). Spearman correlation analysis indicated a significant positive correlation between *P. massiliensis* abundance and ileal Claudin‐1 expression (*p <* 0.05), whereas *B. obeum* abundance was negatively associated with tight junction protein expression across multiple intestinal segments (*p <* 0.05) (Figure 7G).

### 2.8 Influence of Donor Age on Plasma Metabolomic Profiles in Pigs and Identification of Key Metabolites Associated With Intestinal Barrier Function

**FIGURE 8:**
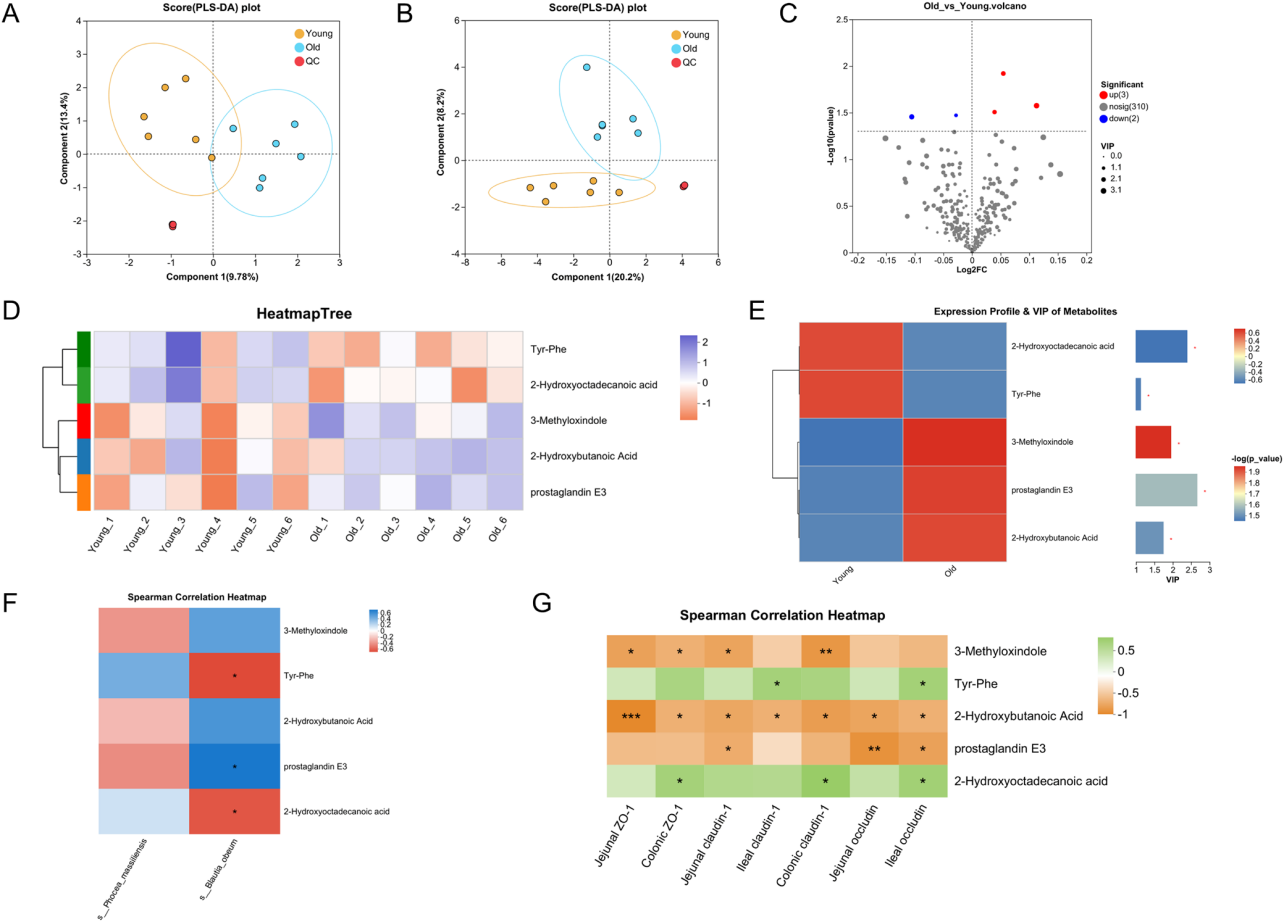
Plasma metabolomic alterations induced by young‐ or elderly‐donor FMT and their associations with key microbial taxa and intestinal barrier proteins. (A, B) Partial least squares–discriminant analysis (PLS‐DA) of cationic and anionic metabolites. (C) Volcano plot of differentially abundant metabolites between old‐FMT and young‐FMT groups. (D) Heatmaptree displaying metabolite clustering and relative abundance. (E) Profiles of significantly altered metabolites. (F) Spearman correlations between key metabolites and *Phocea massiliensis* or *Blautia obeum*. (G) Spearman correlations between key metabolites and intestinal tight junction protein levels. *n* = 6. *p < 0.05; **p < 0.01; ***p < 0.001.

The age of fecal microbiota donors exerted a pronounced impact on plasma metabolite composition in recipient pigs, as well as on the interplay between metabolites, gut microbiota, and intestinal barrier integrity. Partial least squares discriminant analysis (PLS‐DA) demonstrated a distinct separation between the plasma metabolic signatures of pigs transplanted with microbiota from young versus elderly donors under both positive and negative ionization modes (Figure 8A,B). Differential metabolite analysis using volcano plots identified three metabolites that were significantly upregulated and two that were significantly downregulated in the elderly donor group relative to the young donor group (Figure 8C). Heatmap visualization further elucidated the distribution patterns of these five metabolites across individual samples (Figure 8D). Variable importance in projection (VIP) scoring revealed substantial elevations in plasma 3‐methyloxindole, prostaglandin E3, and 2‐hydroxybutanoic acid in pigs receiving elderly donor microbiota, whereas levels of 2‐hydroxyoctadecanoic acid and Tyr‐Phe were markedly diminished (Figure 8E). Spearman correlation analysis highlighted strong associations between microbial taxa and specific metabolites: the two downregulated metabolites exhibited significant negative correlations with *B. obeum* abundance (*p <* 0.05), whereas prostaglandin E3 showed a significant positive correlation with *B. obeum* (*p <* 0.05; Figure 8F). Further analysis of metabolite‐intestinal barrier interactions demonstrated that the three metabolites elevated in the elderly donor group were significantly inversely correlated with tight junction protein expression across multiple intestinal segments (*p <* 0.05). Conversely, the two metabolites reduced in the elderly donor group exhibited significant positive correlations with selected tight junction proteins (*p <* 0.05; Figure 8G). CCK‐8 assay revealed metabolite‐specific effects on the viability of porcine intestinal epithelial cells (IPEC‐1). Tyr‐Phe elicited a dose‐dependent increase in cell viability within the lower concentration range, reaching a maximal response at 10 μM, where viability was significantly elevated compared with the untreated control (*p* < 0.05), followed by a gradual decline at 14 and 20 μM toward basal levels (Figure S2A). Similarly, 2‐hydroxyoctadecanoic acid markedly enhanced IPEC‐1 cell viability, with the most pronounced effect observed at 8 μM (*p* < 0.05); however, this beneficial response was attenuated at higher concentrations, indicating a defined optimal activity window (Figure S2B). In contrast, prostaglandin E3 exerted no overt effect across the tested nanomolar range, as cell viability remained largely comparable to the control group from 1 to 20 nM (Figure S2C). To further evaluate the effects of these key metabolites on intestinal epithelial barrier function, transepithelial electrical resistance (TEER) was measured in IPEC‐1 monolayers following 24 h of metabolite treatment. Compared with the control, Tyr‐Phe (10 μM) significantly increased ΔTEER values (*p* < 0.001), while prostaglandin E3 (PE3, 5 nM) induced a reduction in ΔTEER (*p* < 0.05). Treatment with 2‐hydroxyoctadecanoic acid (2‐HA, 8 μM) showed no significant alteration in TEER in comparison with controls (Figure S3).

### 2.9 Age‐Dependent Modulation of Ileal Gene Expression by Donor Microbiota

**FIGURE 9:**
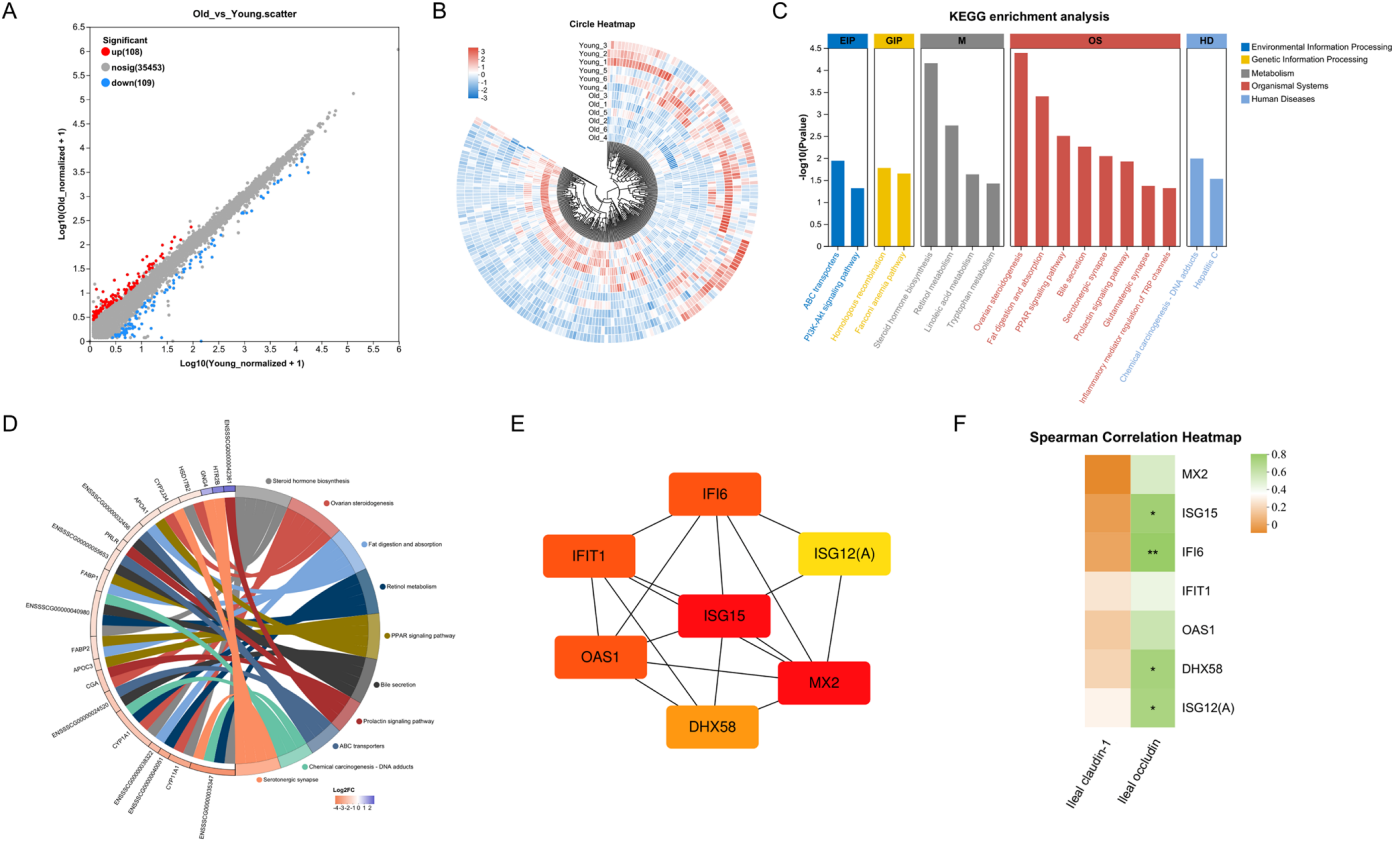
Transcriptomic alterations in the ileal mucosa of pigs receiving young‐ or elderly‐donor FMT. (A) Scatter plot showing 108 genes upregulated and 109 downregulated in the elderly‐donor group compared with the young‐donor group. |Fold change| = 2.0. *p* < 0.05. (B) Circle heatmap illustrating clustering of differentially expressed genes (DEGs) across samples. (C) KEGG pathway enrichment analysis of DEGs, highlighting pathways associated with metabolism, genetic and environmental information processing, and organismal systems. Enrichment significance was determined at an adjusted *p* < 0.05 following Benjamini–Hochberg correction. (D) Network diagram linking the top 10 enriched KEGG pathways to their associated DEGs. (E) Protein–protein interaction (PPI) network revealing seven hub genes (*MX2*, *ISG15*, *IFI6*, *IFIT1*, *OAS1*, *DHX58*, *ISG12(A)*) identified by the maximal clique centrality (MCC) algorithm, all of which were downregulated in elderly‐donor FMT pigs. (F) Correlation analysis showing positive associations between several hub genes and intestinal tight junction protein expression. *n* = 6. *p < 0.05; **p < 0.01.

To evaluate the impact of donor age on host intestinal gene expression, transcriptomic profiling was performed on ileal mucosa from pigs transplanted with fecal microbiota from young or elderly donors. Differential expression analysis revealed that 108 genes were significantly upregulated and 109 genes were significantly downregulated in the elderly donor group compared with the young donor group (Figure 9A). Clustering analysis based on these differentially expressed genes (DEGs) is illustrated in the Circle Heatmap (Figure 9B). KEGG pathway enrichment analysis of these DEGs demonstrated significant enrichment in pathways related to steroid hormone biosynthesis, ovarian steroidogenesis, fat digestion and absorption, retinol metabolism, PPAR signaling pathway, bile secretion, and etc., highlighting potential functional consequences of donor age‐dependent microbial modulation (Figure 9C). The associations between the top 10 significantly enriched KEGG pathways (*p <* 0.05) and their corresponding DEGs are presented in Figure 9D. Protein–protein interaction (PPI) networks constructed from DEGs were analyzed using the cytoHubba plugin in Cytoscape, employing the maximal clique centrality (MCC) algorithm to identify hub genes. The top seven hub genes, ranked by MCC score, included *MX2*, *ISG15*, *IFI6*, *IFIT1*, *OAS1*, *DHX58*, and *ISG12(A)* (Figure 9E). Notably, all seven hub genes were significantly downregulated in the ileal mucosa of pigs receiving elderly donor microbiota compared with those receiving young donor microbiota (Table S3). Correlation analysis between DEGs and intestinal tight junction proteins revealed that expression levels of *ISG15*, *IFI6*, *OAS1*, *DHX58*, and *ISG12(A)* were significantly positively associated with ileal tight junction protein expression (*p <* 0.05; Figure 9F). These findings suggest that age‐related differences in donor microbiota can modulate host mucosal transcriptomic profiles, potentially influencing intestinal barrier integrity via regulation of genes associated with immune responses and metabolic pathways.

## 3 Discussion

With the rapid acceleration of global population aging, elucidating the mechanisms underlying age‐associated intestinal decline has become a critical priority for promoting healthy longevity. In the present study, we established a porcine model via FMT, which not only recapitulated the physiological and pathological features of the aging intestine but also enabled a systematic dissection of the determinants shaping intestinal health. This approach provided an experimental framework for the rational design of evidence‐based dietary and microbiota‐targeted interventions. Our findings demonstrated that the chronological age of FMT donors exerts a decisive influence on intestinal homeostasis. Specifically, transplantation of microbiota from aged donors disrupted intestinal barrier integrity through remodeling of microbial community architecture, perturbation of host metabolic signatures, and modulation of gene expression programs within the intestinal mucosa. Collectively, these results establish a link between age‐dependent microbiota shifts and host intestinal dysfunction, thereby laying a foundation for leveraging FMT and microbiome‐informed strategies in geriatric health promotion.

Although pigs share a high degree of anatomical similarity in their gastrointestinal tract with humans, host species remains a fundamental determinant of gut microbiota composition. Previous studies have shown that even under identical environmental and dietary conditions, distinct species harbor divergent microbial communities (Yao et al. 2024). Consequently, xenotransplantation of human‐derived fecal microbiota into pigs provides a more accurate platform to reconstruct and model human‐specific gut microbial structures. Consistent with this notion, our study demonstrated that despite being raised under uniform husbandry and dietary conditions, pigs transplanted with human fecal microbiota exhibited marked differences in gut microbial composition compared to conventional pigs. Moreover, pigs colonized with aged donor microbiota exhibited impaired intestinal homeostasis, characterized by a reduced microbiota health index and an elevated dysbiosis index, which aligns with previous findings (Malik et al. 2023). In terms of microbiota composition, prior studies have reported that elderly individuals (≥ 65 or 70 years) typically harbor a higher relative abundance of Bacteroidetes and a concomitant reduction in Firmicutes compared to younger adults (Claesson et al. 2011; Odamaki et al. 2016). In agreement with these findings, we observed that pigs colonized with microbiota from aged donors exhibited a lower mean relative abundance of Firmicutes and a higher abundance of Bacteroidetes. Interestingly, our analysis revealed that pigs receiving microbiota from young donors harbored higher levels of *Phocea massiliensis*, whereas *Blautia obeum* emerged as a signature species in pigs transplanted with aged donor microbiota. *P. massiliensis* , a strict anaerobic Gram‐negative bacillus present in the intestinal tract of both humans and animals (Ndongo et al. 2016), also shows age‐associated decline in murine models (Ahn et al. 2025). Although its functional role remains largely undefined, murine studies have reported a negative correlation between the frailty index, an integrative measure of adverse aging outcomes (Schultz et al. 2020), and the abundance of *P. massiliensis* (Ke et al. 2021). This suggests that the reduction of *P. massiliensis* in elderly individuals may serve as a microbial indicator of increased susceptibility to morbidity and mortality. Conversely, *B. obeum*, a Gram‐positive anaerobe, has been implicated as a potential risk factor in several diseases. For instance, it has been shown to exacerbate colitis in mice (Lee et al. 2023), indicating its pathogenic potential in inflammatory contexts. Moreover, an increased abundance of *B. obeum* has been associated with decreased fasting C‐peptide levels, suggesting a potential association with diabetes risk (Vatanen et al. 2024). Collectively, these observations suggest that the enrichment of *B. obeum* in the elderly gut may predispose individuals to age‐related disease susceptibility.

With advancing age, intestinal stem cell function progressively declines, potentially contributing to structural and functional impairments in the intestinal tract. Nevertheless, evidence regarding villus height, crypt depth, and the villus‐to‐crypt ratio is conflicting, with some studies reporting clear alterations and others detecting little to no change, often in an intestinal segment–specific manner. For instance, an experimental study in canines demonstrated that jejunal villus height was markedly reduced in aged compared to young dogs (Kuzmuk et al. 2005). In our study, we observed that pigs receiving fecal microbiota from elderly donors exhibited a significant reduction in ileal villus height compared to those transplanted with microbiota from young donors. Such reductions in villus height may partially account for the impaired nutrient absorption frequently reported in older adults. Indeed, previous studies have documented age‐associated declines in the absorption of carbohydrates, lipids, amino acids, minerals, and vitamins (Woudstra and Thomson 2002). Consistent with these findings, transcriptomic profiling of ileal mucosa from pigs colonized with elderly versus young donor microbiota revealed differential gene expression enriched in pathways related to lipid digestion and absorption, suggesting that age‐dependent transcriptional reprogramming may represent an additional mechanistic determinant of impaired lipid assimilation.

The intestinal mechanical barrier, formed by epithelial cells, plays an indispensable role in preventing the invasion of harmful substances and pathogens while maintaining intestinal homeostasis (Chen et al. 2025). Aging, however, is associated with increased intestinal permeability and a concomitant reduction in tight junction protein expression (Branca et al. 2019). In this study, pigs receiving microbiota from elderly donors exhibited significantly lower levels of tight junction proteins (ZO‐1, Claudin‐1, and Occludin) across multiple intestinal segments compared with those receiving microbiota from young donors. This reduction was associated with elevated DAO activity, indicative of enhanced intestinal permeability. Such alterations can be partly attributed to age‐associated microbial dysbiosis, a phenomenon that has also been demonstrated in murine models (Jing et al. 2025). Notably, our analysis further revealed correlations between the abundance of specific bacterial taxa and the expression levels of tight junction proteins in distinct intestinal segments, thereby offering novel microbial insights into the potential factors underlying barrier dysfunction during aging.

Among the five discriminatory metabolites identified in this study, Tyr‐Phe, 2‐hydroxyoctadecanoic acid, and prostaglandin E3 were closely associated with *B. obeum* and displayed contrasting relationships with intestinal tight junction proteins. Circulating Tyr‐Phe and 2‐hydroxyoctadecanoic acid concentrations were lower in pigs receiving elderly‐donor microbiota and correlated positively with several tight junction proteins. By contrast, prostaglandin E3 was elevated and showed inverse associations with these barrier‐related markers. This reciprocal pattern suggests that elderly‐donor FMT may reshape the systemic metabolic milieu in a manner linked to compromised intestinal barrier homeostasis. Tyr‐containing dipeptides possess antioxidant activity, largely attributable to the radical‐scavenging capacity of the phenolic moiety of tyrosine (Chen et al. 2020), whereas 2‐hydroxyoctadecanoic acid is a long‐chain 2‐hydroxy fatty acid involved in lipid metabolism and membrane organization (Foulon et al. 2005). These reported biological properties suggest that both Tyr‐Phe and 2‐hydroxyoctadecanoic acid probably contribute to the maintenance of epithelial cell survival and renewal, which are essential for maintaining mucosal integrity. Consistent with this possibility, in vitro validation using IPEC‐1 cells demonstrated that Tyr‐Phe supplementation at a specific dosage resulted in an increase in both cell viability and TEER, providing functional evidence for its potential role in enhancing intestinal epithelial barrier. Although 2‐hydroxyoctadecanoic acid did not alter TEER under the tested conditions, it promoted IPEC‐1 cell viability, indicating its effects may be associated with epithelial cell maintenance. In contrast, prostaglandin E3 exerted no apparent influence on cell viability while reducing TEER, which demonstrates a potential detrimental effect on intestinal epithelial barrier independent of cytotoxicity. This observation is in agreement with a previous finding that prostaglandin E3 increased paracellular permeability in Caco‐2 monolayers through EP1‐ and EP4‐mediated signaling and reorganization of tight junction‐associated structures (Rodriguez‐Lagunas et al. 2013). Collectively, these observations support a model in which elderly‐donor microbiota are associated with lower levels of metabolites potentially favorable to epithelial maintenance, together with increased prostaglandin E3, which may promote permeability. However, the relationships involving *B. obeum* remain correlative, and direct mechanistic links will require targeted metabolite interventions and dedicated epithelial barrier assays.

Transcriptomic analysis revealed that DEGs between pigs receiving elderly versus young donor microbiota were enriched in pathways including steroid hormone biosynthesis, ovarian steroidogenesis, retinol metabolism, PPAR signaling, and bile secretion. These pathway‐level alterations indicate that aging‐associated microbiota may reshape host intestinal physiology through coordinated effects on lipid‐derived endocrine signaling, retinoid metabolism, nuclear receptor activity, and bile acid homeostasis. The decline in steroid hormone biosynthesis with aging can contribute to mitochondrial dysfunction in steroid‐responsive tissues (Velarde 2014). Similarly, age‐associated suppression of ovarian steroidogenesis has been demonstrated in murine models (Vo et al. 2025). Although ovarian steroidogenesis is not an intestine‐specific pathway, genes mapped to this pathway often overlap with steroidogenic and lipid‐metabolic enzymes; therefore, its enrichment in ileal mucosa may reflect altered local lipid handling or steroid‐related metabolic signaling rather than canonical ovarian endocrine activity. The enrichment of retinol metabolism observed in our study is also consistent with findings from studies of hepatic aging (Zhang et al. 2021). Importantly, retinoid metabolism is closely linked to intestinal epithelial homeostasis and mucosal immune regulation (Cao et al. 2022; Grizotte‐Lake et al. 2018; Jijon et al. 2018). Thus, altered retinol metabolism in recipients of microbiota from elderly donors may have functional implications for epithelial renewal. Alterations in the PPAR signaling pathway have been strongly linked to aging (Li et al. 2019), given its pivotal role in regulating energy metabolism and oxidative stress responses (Erol 2007). Inhibition of PPAR signaling has also been shown to impair mitochondrial function (Liu et al. 2013). Furthermore, enrichment of bile secretion‐related genes suggests age‐associated impairment of bile secretion, primarily driven by dysregulated bile acid metabolism and signaling (Jin et al. 2024, 2025). This is relevant because bile acids are not only digestive molecules but also microbiota‐modified signaling metabolites that regulate intestinal epithelial renewal, permeability, and mucosal immune homeostasis (Calzadilla et al. 2022; Sorrentino et al. 2020).

In the ileal mucosa, our transcriptomic analysis identified several hub genes, including *MX2*, *ISG15*, *IFI6*, *IFIT1*, *OAS1*, *DHX58*, and *ISG12(A)*. A notable feature of these hub genes is that they do not represent unrelated immune markers; rather, they form a coherent interferon‐stimulated gene (ISG) module downstream of Type I and Type III interferon signaling (Pott and Stockinger 2017; Schoggins and Rice 2011). This pattern suggests that recipients of elderly donor microbiota exhibited a reduced epithelial interferon‐responsive antiviral state. Among them, MX2, IFI6, IFIT1, OAS1, and DHX58 are key players in antiviral defense (Harioudh et al. 2024; Kirby et al. 2024; Meng et al. 2022; Reynaud et al. 2015; Zhao et al. 2025). Mechanistically, MX2 functions as an interferon‐induced restriction factor that blocks viral replication at post‐entry steps (Goujon et al. 2013); IFIT1 recognizes non‐self viral RNA species, including RNAs with abnormal cap modification or 5′‐triphosphate structures, thereby suppressing viral translation (Diamond 2014); OAS1 activates the OAS/RNase L antiviral pathway to degrade viral and cellular RNAs during infection (Silverman 2007); and DHX58/LGP2 modulates RIG‐I‐ and MDA5‐dependent viral RNA sensing (Gillich et al. 2023). Therefore, the simultaneous downregulation of MX2, IFIT1, OAS1, and DHX58 implies attenuation at multiple levels of the epithelial antiviral cascade, including viral sensing, RNA restriction, and downstream effector activity. Notably, these genes were significantly downregulated in pigs transplanted with microbiota from elderly donors, suggesting compromised antiviral capacity. This interpretation is particularly relevant in the intestine, where tonic interferon signaling is not merely a response to overt infection but also contributes to basal mucosal preparedness (Pott and Stockinger 2017). Commensal bacterial microbiota can stimulate localized IFN‐λ‐dependent ISG expression in intestinal epithelial cells, thereby establishing preemptive antiviral defense within discrete epithelial regions (Van Winkle et al. 2022). Thus, elderly donor microbiota may either provide weaker microbial cues for maintaining this homeostatic ISG tone or generate metabolites/inflammatory signals that dampen epithelial interferon responsiveness. ISG15, which is upregulated in intestinal epithelial cells during IBD and contributes to immune regulation (Ostvik et al. 2020), was reduced in elderly donor recipients, potentially attenuating mucosal immune responses under inflammatory conditions. ISG15 is especially important because it acts both as a ubiquitin‐like modifier through ISGylation and as an extracellular immunomodulatory molecule; consequently, reduced ISG15 may weaken antiviral restriction while also altering cytokine crosstalk between epithelial and immune cells (Perng and Lenschow 2018). Deficiency of ISG15 has been associated with exacerbated inflammation (Lenschow et al. 2007), consistent with our observation that reduced ISG15 expression was accompanied by decreased Occludin levels in the ileal mucosa. This association provides a plausible mechanistic link between suppression of the interferon/ISG module and impaired epithelial barrier integrity. IFI6 is implicated in preventing apoptosis and maintaining mitochondrial homeostasis (Jia et al. 2020; Qi et al. 2015), both critical for sustaining epithelial integrity and barrier function. Its positive correlation with Occludin expression in our study supports this role. The inclusion of ISG12(A), also referred to as IFI27 in several species, further connects the ISG signature with mitochondrial regulation and epithelial cell fate, because ISG12 family proteins have been implicated in mitochondria‐associated apoptosis and antiviral responses (Shojaei and McLean 2025). In the aging intestine, where epithelial renewal, mitochondrial quality control, barrier integrity, microbiota composition, and immune homeostasis are frequently compromised (Hohman and Osborne 2022), coordinated suppression of IFI6 and ISG12(A) may reduce the ability of epithelial cells to balance survival, apoptosis, and antiviral defense under microbial or inflammatory stress. Although our findings suggest potential links between these hub genes and barrier function during aging, further experimental validation is required to establish their mechanistic contributions in elderly populations and under specific pathological conditions. Future studies should determine whether aged microbiota directly suppress epithelial IFN‐I/IFN‐III signaling, whether supplementation with young donor microbiota or defined microbial metabolites restores ISG expression, and whether manipulation of the IFN–ISG axis can rescue Occludin expression and ileal barrier function. Such work would help clarify whether the ISG module identified here is merely a transcriptomic marker of microbiota‐driven aging or a causal mediator linking aged microbiota to impaired mucosal antiviral defense and epithelial barrier dysfunction.

An important strength of the present study is the use of a porcine recipient model rather than a conventional rodent model. Compared with rodents, pigs more closely approximate humans with respect to omnivorous feeding behavior, gastrointestinal scale and architecture, intestinal motility and transit characteristics, and the predominance of colonic rather than cecal fermentation, all of which are relevant determinants of microbial ecological niches and host–microbiota interactions (Gonzalez et al. 2015; Rose et al. 2022; Ziegler et al. 2016). These features render the porcine intestine a particularly informative platform for investigating the integrated consequences of human‐derived FMT on microbial community structure, epithelial barrier integrity, mucosal transcriptional programs, and metabolic phenotypes. Notably, a direct comparison of human microbiota‐associated piglets and mice under matched experimental conditions demonstrated that mature human donor microbiota established more efficiently and persistently in piglets (Aluthge et al. 2020), which is especially pertinent to the present human donor‐derived FMT paradigm. Thus, while rodent models remain indispensable for high‐throughput and genetically targeted mechanistic studies, the porcine model provides a valuable translational bridge between reductionist rodent experiments and human intestinal biology.

However, several considerations should be acknowledged when interpreting the present findings. By integrating a human‐derived FMT pig model with histological assessment, multi‐omics profiling, and correlation analyses, this study provides a systematic characterization of the associations between microbiota derived from elderly donors and host intestinal architecture, barrier function, metabolic profiles, and mucosal transcriptional responses. Nevertheless, because the current dataset is primarily based on comparative and correlation‐based evidence, the specific causal contributions of individual microbial taxa, metabolites, and host hub genes remain to be further delineated. In addition, the relatively short observation period and moderate sample size may have limited the extent to which the observed effects can be generalized across broader biological contexts. Future studies incorporating longer‐term follow‐up, expanded cohorts, and targeted interventions directed at key microbial species, metabolites, and host pathways will help refine the causal framework underlying these host–microbiota interactions and further substantiate the mechanistic relevance of the signatures identified here.

## 4 Conclusion

In summary, this study employed a porcine fecal microbiota transplantation model to systematically characterize the multifaceted effects of elderly donor‐derived microbiota on host intestinal homeostasis, barrier function, metabolic profiles, and gene expression. Compared with microbiota from young donors, elderly donor microbiota not only induced marked dysbiosis and reshaped microbial community structure but also altered host metabolite levels, reduced ileal villus height, increased intestinal permeability, and downregulated the expression of tight junction proteins. Transcriptomic profiling further revealed DEGs and enriched pathways in the intestinal mucosa under the influence of elderly microbiota, as well as the identification of hub genes with potential functional significance. These findings provide experimental support for a close association between age‐related microbiota alterations and intestinal barrier dysfunction. This work expands our understanding of microbiota–host interactions in intestinal aging and offers candidate directions for future microbiota‐targeted nutritional or therapeutic studies. Further research is needed to validate the causal roles of specific microbial taxa and metabolites and to assess their translational relevance in age‐associated disorders.

## 5 Methods

### 5.1 Materials

Hematoxylin and eosin (H&E) staining solution, CCK‐8 kit, DMEM/F12 medium, and fetal bovine serum (FBS) were purchased from I‐presci Scientific Co. Ltd. (Beijing, China). Alcian Blue staining kit was obtained from Vector Laboratories (CA, USA). Diamine oxidase (DAO) assay kit was purchased from Suzhou Grace Biotechnology Co. Ltd. (Suzhou, China). ELISA kits for porcine claudin‐1, occludin, and ZO‐1 were purchased from Xiamen LCS Biotechnology Co. Ltd. (Xiamen, China). Tyr‐Phe and 2‐hydroxyoctadecanoic acid were purchased from Aladdin Bio‐Chem Technology Co. Ltd. (Shanghai, China), and 17‐trans prostaglandin E3 was supplied by Yeasen Biotechnology Co. Ltd. (Shanghai, China).

### 5.2 Animals and Experimental Design

A total of 18 male Bama miniature pigs, 2 months of age, with an average initial body weight of 3.60 ± 0.13 kg, were selected for this experiment. The pigs were randomly allocated into three groups (*n* = 6 per group): the control group, the young donor fecal microbiota transplantation group (Young group), and the elderly donor fecal microbiota transplantation group (Old group). To deplete the gut microbiota, pigs received a daily oral gavage of a broad‐spectrum antibiotic cocktail (vancomycin (250 mg/kg), ampicillin (500 mg/kg), neomycin (500 mg/kg), and metronidazole (250 mg/kg)) for 3 consecutive days. Thereafter, omeprazole (20 mg/kg) was administered to reduce gastric acid, followed by alternate‐day gavage of human fecal microbiota suspensions (4 × 10^9^ CFU, derived from either young or elderly donors) over an 8‐day period. Fecal microbiota from young and elderly people were obtained from fecal samples collected from healthy individuals aged 22–29 years and 70–85 years, respectively. We recruited five donors from both the young and elderly groups, with each donor providing two separate fecal samples. Thus, each group consisted of 10 samples in total. The experimental workflow is illustrated in Figure 1A. After 7 days of microbial colonization, biological samples were collected.

The pigs were housed under controlled environmental conditions, with the ambient temperature maintained at 23°C–26°C, relative humidity regulated at 40%–50%, and a 12‐h light/dark cycle implemented. The diet composition and nutrient levels are provided in Table S1, in accordance with the recommendations of the National Research Council (NRC 2012). Feed and water were provided ad libitum. Feeding occurred twice daily at 10:30 a.m. and 5:00 p.m. Pens and equipment were thoroughly cleaned and disinfected before use, and daily maintenance procedures included the removal of fecal and urinary waste.

At the end of the trial, 12 pigs from both the Young and Old groups were subjected to overnight fasting and subsequently anesthetized using carbon dioxide inhalation. Blood samples (10 mL) were collected via jugular venipuncture into anticoagulant‐treated tubes after the pigs were anesthetized. Subsequently, the animals were humanely euthanized, and after confirmation of the complete cessation of vital signs, the abdominal cavity was opened for tissue collection. The duodenum, jejunum, ileum, and colon were carefully excised for further analysis. Colonic contents were collected. For histological analysis, approximately 2 cm mid‐segments were excised from each intestinal section, rinsed with pre‐cooled phosphate‐buffered saline (PBS), and fixed in 4% paraformaldehyde solution. Remaining intestinal tissues were longitudinally opened, rinsed, and mucosal layers were scraped, snap‐frozen in liquid nitrogen, and stored at −80°C.

### 5.3 Hematological Analysis

Fresh blood samples were collected by cranial vena cava puncture and immediately transferred into evacuated anticoagulant tubes containing EDTA‐K_2_. The tubes were gently inverted several times to ensure thorough mixing with the anticoagulant and to prevent clot formation. Following collection, the samples were immediately placed in a refrigerated container maintained at 4°C and transported to the laboratory within 2 h for subsequent processing. Prior to routine hematological analysis, the samples were allowed to equilibrate to room temperature (approximately 20°C–25°C) and were gently inverted again to ensure homogeneity. The hematological parameters were then determined using an automated hematology analyzer (Sysmex XN‐1000V, Sysmex, Japan). Parameters measured included red blood cell count (RBC), hematocrit (HCT), hemoglobin (HGB), mean corpuscular volume (MCV), mean corpuscular hemoglobin (MCH), mean corpuscular hemoglobin concentration (MCHC), red cell distribution width‐coefficient of variation (RDW‐CV), nucleated red blood cell percentage (NRBC%), nucleated red blood cell count (NRBC), white blood cell count (WBC), neutrophil percentage (NEUT%), neutrophil count (NEUT), lymphocyte percentage (LYMPH%), monocyte percentage (MONO%), eosinophil percentage (EO%), basophil percentage (BASO%), basophil count (BASO, 10^9^/L), lymphocyte count (LYMPH), monocyte count (MONO), eosinophil count (EO), basophil count (BASO), platelet count (PLT), mean platelet volume (MPV), plateletcrit (PCT), platelet distribution index (PLT‐I), and platelet large cell ratio (P‐LCR).

### 5.4 Plasma Biochemical Analysis

The collected anticoagulated blood samples were centrifuged at 3000 rpm for 15 min at 4°C. The upper plasma layer was then carefully aspirated and transferred into fresh centrifuge tubes. After aliquoting, the plasma samples were stored at −80°C in an ultra‐low‐temperature freezer until further analysis. Plasma biochemical parameters were assessed using an automated biochemical analyzer (Cobas 6000 c501, Roche, Switzerland). Measurements included alanine aminotransferase (ALT), aspartate aminotransferase (AST), total protein (TP), albumin (ALB), globulin (GLB), albumin/globulin ratio (A/G), γ‐glutamyltransferase (GGT), creatine kinase (CK), glucose (GLU), urea (UREA), creatinine (CREA), total cholesterol (CHOL), triglycerides (TG), high‐density lipoprotein cholesterol (HDL‐C), low‐density lipoprotein cholesterol (LDL‐C), total bile acids (TBA), fructosamine (FRU), and non‐esterified fatty acids (NEFA).

### 5.5 Diamine Oxidase Assay

Diamine oxidase (DAO) activity was measured using a commercial kit (Grace Biotechnology, Suzhou, China). Blood samples were centrifuged at 1250 *g* for 15 min at 4°C to obtain plasma. DAO content was determined using the 4‐aminoantipyrine (4‐AAP) colorimetric method at 510 nm, following the instructions provided by the manufacturer and expressed as ΔOD_510_/min/mL.

### 5.6 Hematoxylin and Eosin (H&E) Staining

Intestinal tissues were rinsed thoroughly and fixed in 4% paraformaldehyde for 48 h, followed by storage in 70% ethanol. Fixed samples were trimmed and subjected to graded ethanol dehydration (90%, 95%, and 100%, 1 h each), cleared in xylene I (10 min) and xylene II (5 min), and subsequently infiltrated with paraffin. Samples were immersed sequentially in xylene‐paraffin (1:1, 60°C, 2 h), paraffin I (60°C, 2 h), and paraffin II (overnight). After embedding, paraffin blocks were sectioned at 5 μm using a rotary microtome, expanded in a 42°C water bath, and mounted onto glass slides. Slides were dried at 42°C for 24 h, deparaffinized in xylene (15 min), rehydrated through graded ethanol (100%, 95%, 70%; 5 min each), and rinsed in distilled water. Sections were stained with hematoxylin for 2 min until nuclei appeared blue, rinsed in tap water three times (5 min each), counterstained with eosin for 40 s, and washed twice in running tap water (5 min each). Slides were dehydrated through graded ethanol (70%, 95%, 100%; 5 s, 3 min, and 5 min, respectively), cleared in xylene I and II (5 min each), and mounted with neutral balsam. Histological features were examined and imaged under a light microscope. For each section, five non‐overlapping microscopic fields were uniformly and randomly selected along the longitudinal axis of the tissue, avoiding the edges of the section and villi with oblique orientation. In each field, the height of two randomly selected, intact, vertically oriented intestinal villi and the corresponding crypt depth were measured. Thus, a total of 10 sets of measurements were obtained for each section. The mean values of these 10 measurements were used as the representative villus height and crypt depth for the corresponding intestinal segment of each sample, and the villus height‐to‐crypt depth ratio (V/C) was subsequently calculated. All measurements were performed in a blinded manner by the same observer, and calibration and data recording were conducted using NIS‐Elements D image analysis software (Nikon, Japan).

### 5.7 Alcian Blue Staining

Paraffin‐embedded intestinal sections were deparaffinized, rehydrated, and immersed in 3% acetic acid for 3 min to facilitate staining. Alcian Blue solution was applied, and sections were incubated in a humidified chamber at room temperature for 30 min. After brief rinsing in 3% acetic acid (~10 s) to remove excess dye, sections were washed with distilled water. Nuclear Fast Red counterstaining was performed for 5 min, followed by washing in distilled water for 5 min. Slides were mounted with neutral balsam and imaged under a microscope. To assess mucin content, 10 well‐preserved crypts containing acidic mucins were randomly selected per section, and the number of Alcian Blue–positive granules was quantified.

### 5.8 Enzyme‐Linked Immunosorbent Assay

Tissue samples from each intestinal segment were pulverized into a fine powder. For protein extraction, 450 μL of pre‐chilled PBS containing protease inhibitors and phenylmethylsulfonyl fluoride (PMSF) was added to every 50 mg of tissue powder. Samples were homogenized on ice using an ultrasonic cell disruptor with 5‐s intermittent pulses. The probe was thoroughly rinsed with purified water between samples to prevent cross‐contamination. Following five cycles of sonication, the homogenates were subjected to centrifugation at 12,000 × *g* for 30 min at 4°C, after which the resulting supernatants were carefully collected, and protein concentrations were determined using the BCA assay before storage at −80°C pending subsequent analysis. ELISA assays were performed using commercial kits following the manufacturer's instructions. All reagents and samples were equilibrated to room temperature (25°C–28°C) for 60 min prior to use. Standards, samples, and blanks were set up in triplicate. Standard wells received 50 μL of serially diluted standards, sample wells received 50 μL of sample, and blank wells received 50 μL of diluent. HRP‐conjugated detection antibody (100 μL) was added to each well, sealed with adhesive film, and incubated at 37°C for 45 min. Plates were washed five times with wash buffer, and substrate solutions A and B (50 μL each) were added sequentially. After incubation at 37°C in the dark for 15 min, 50 μL stop solution was added. Optical density (OD) was measured at 450 nm within 15 min using a microplate reader.

### 5.9 Full‐Length 16S rRNA Gene Sequencing

Genomic DNA was extracted from samples using a commercial kit. DNA concentration and purity were determined using a NanoDrop 2000 spectrophotometer, and integrity was assessed by 1% agarose gel electrophoresis. Full‐length bacterial 16S rRNA gene sequencing targeting the V1–V9 region was performed using primers 27F (5′‐AGRGTTYGATYMTGGCTCAG‐3′) and 1492R (5′‐RGYTACCTTGTTACGACTT‐3′). Equimolar purified PCR products were pooled to construct sequencing libraries with the SMRTbell kit, followed by sequencing on the PacBio Sequel IIe platform. Raw reads were processed using SMRTLink to obtain high‐quality HiFi reads, and sequences shorter than 1000 bp or longer than 1800 bp were removed. Denoising was conducted using the DADA2 plugin in QIIME2 to generate amplicon sequence variants (ASVs). Bioinformatic analyses, including α‐diversity estimation, principal coordinate analysis (PCoA), and PERMANOVA, were conducted on the Majorbio Cloud platform. Linear discriminant analysis effect size (LEfSe) was applied to identify taxa with significant differential abundance between groups, with an LDA score threshold of > 2.0.

### 5.10 Transcriptomic Analysis of Intestinal Mucosa

Total RNA from intestinal tissues was extracted using TRIzol reagent. RNA libraries were prepared with the Hieff NGS Ultima Dual‐mode RNA Library Prep Kit (Yeasen). Poly(A) + mRNA was enriched using Oligo(dT) beads, followed by fragmentation, cDNA synthesis, end repair, adaptor ligation, PCR amplification, and single‐strand circularization. Library quality was assessed using Qubit 4.0 for quantification, Agilent Bioanalyzer for insert size distribution, and qRT‐PCR for accurate library concentration. Libraries were sequenced on the Illumina NovaSeq 6000 platform. Raw reads were filtered with fastp, and clean reads were aligned to the reference genome using HISAT2. Transcript assembly was performed with StringTie, and gene expression was quantified using FeatureCounts, normalized to fragments per kilobase of transcript per million mapped reads (FPKM). DEGs were identified using DESeq2 based on the criteria of adjusted *p* < 0.05 and |log_2_ fold change| ≥ 1, corresponding to an absolute fold change of ≥ 2.0. Functional enrichment analyses were conducted with clusterProfiler based on KEGG annotation (adjusted *p* < 0.05). Network topology and hub gene identification were performed in Cytoscape (v3.10.2) using the cytoHubba plugin with the maximal clique centrality (MCC) algorithm. The top seven genes ranked by MCC were designated as hub genes.

### 5.11 Metabolomic Analysis of Plasma

Plasma metabolites were analyzed by Metware Biotechnology (Wuhan, China). Plasma samples stored at −80°C were thawed on ice and vortexed for 10 s. Aliquots of 50 μL were extracted with 300 μL of extraction buffer, vortexed for 3 min, and centrifuged at 12,000 rpm for 10 min at 4°C. A 200 μL supernatant was collected, incubated at −20°C for 30 min, centrifuged again (12,000 rpm, 3 min, 4°C), and 180 μL of supernatant was subjected to LC–MS analysis. UHPLC‐Q Exactive HF‐X equipped with an ACQUITY HSS T3 column (2.1 × 100 mm, 1.8 μm) was used for chromatographic separation. Mass spectrometry was conducted with an electrospray ionization (ESI) source in both positive and negative ion modes. Quality control (QC) samples, prepared by pooling equal aliquots from all samples, were injected every 5–15 test samples to monitor system stability. Differential metabolites were identified based on variable importance in projection (VIP > 1) and *p <* 0.05. Data were log‐transformed, mean‐centered, and validated using permutation testing to avoid overfitting. Identified metabolites were annotated against the KEGG compound database and mapped to KEGG pathways for functional interpretation.

### 5.12 Cell Culture and CCK‐8 Assay

IPEC‐1 cells were maintained in DMEM/F12 medium supplemented with 10% fetal bovine serum (FBS) and 1% penicillin–streptomycin solution under standard culture conditions at 37°C in a humidified atmosphere containing 5% CO_2_. Cells in the logarithmic growth phase were seeded into 96‐well plates at a density of 9 × 10^3^ cells per well and allowed to adhere completely. The culture medium was then removed, and the cells were serum‐starved in serum‐free DMEM/F12 medium for 6 h. Thereafter, the medium was replaced with DMEM/F12 containing 1% FBS and serial concentrations of Tyr‐Phe, 2‐hydroxyoctadecanoic acid, or prostaglandin E3. Following 24 h of metabolite exposure, 10 μL of CCK‐8 reagent was added to each well containing 100 μL of DMEM/F12 medium, and the plates were incubated for an additional 40 min at 37°C. Absorbance was subsequently measured at 450 nm using a microplate reader, and relative cell viability was calculated based on the absorbance values.

For transepithelial electrical resistance (TEER) measurement, IPEC‐1 cells were seeded at a density of 5 × 10^4^ cells per insert onto 6.5 mm diameter Transwell inserts in 24‐well plates. After 18 h of incubation, the baseline TEER value was measured and defined as 0 h. Cells were then treated with Tyr‐Phe (10 μM), 2‐hydroxyoctadecanoic acid (2‐HA, 8 μM), or prostaglandin E3 (PE3, 5 nM) for 24 h, followed by TEER measurement. TEER values were recorded using a Millicell ERS2 epithelial voltohmmeter. The resistance values of blank inserts without cells were measured and subtracted from all readings. The corrected TEER values were calculated by multiplying the resistance difference by the effective membrane surface area (0.33 cm^2^). The changes in TEER (ΔTEER) were calculated as the difference between the values measured at 24 h and 0 h.

### 5.13 Statistical Analysis

Data are presented as mean ± standard deviation (SD) unless otherwise indicated. One‐way analysis of variance (ANOVA) followed by Duncan's multiple range test was performed using SPSS 26.0. Two‐group comparisons were analyzed with a two‐tailed unpaired Student's *t*‐test using GraphPad Prism (v9.5). For cell culture‐related experiments involving multiple group comparisons, statistical significance was assessed using one‐way ANOVA followed by Dunnett's post hoc test by GraphPad Prism (v9.5). Statistical significance was defined as *p <* 0.05, while *p <* 0.01 and *p <* 0.001 indicated highly significant differences.

## Author Contributions

J.W. performed the experiments, handled animals, analyzed the data, and wrote the original draft. L.L. contributed to writing the original draft and performing the experiments. L.M., B.L. and Q.Z. assisted with sample assays. Z.T. handled animals. J.W., L.M., Y.W., Q.F., L.R., Z.Z., Y.X., A.Y., and S.D. collected samples. Z.Z., Y.H., Y.Z., Y.X, Y.Y., and Z.W. participated in experimental design and discussed the data. Y.J. designed the experiments, analyzed omics data, and revised the manuscript. All authors read and approved the final manuscript.

## Funding

This work was supported by the National Key R&D Program of China (nos. 2022YFF1100104, 2022YFD1300501, and 2022YFF1100102).

## Ethics Statement

All experimental procedures strictly adhered to ethical guidelines and were approved by the Human Research Ethics Committee of China Agricultural University (approval no. CAUHR‐20240304) and the Animal Welfare and Experimental Ethics Committee of China Agricultural University (approval no. AW41304202‐1‐4).

## Conflicts of Interest

The authors declare no conflicts of interest.

## Supporting information


**Table S1:** Effects of fecal microbiota transplantation from young or elderly donors on the blood routine indicators of pigs.
**Table S2:** Impacts of human fecal microbiota transplantation on blood biochemical parameters in pigs.
**Table S3:** Fold change of the 7 hub genes identified.
**Figure S1:** ACE‐based rarefaction curves of microbial communities following antibiotics treatment and fecal microbiota transplantation. (A) ACE rarefaction curves of control and antibiotics‐treated groups. (B) ACE rarefaction curves of recipients after FMT from young or elderly donors. Each curve represents an individual sample. Abx, antibiotics. ACE, Abundance‐based Coverage Estimator.
**Figure S2:** Effects of treatment with different concentrations of three key metabolites on the viability of porcine intestinal epithelial (IPEC‐1) cells. IPEC‐1 cells were treated with serial concentrations of Tyr‐Phe (A), 2‐hydroxyoctadecanoic acid (B), or prostaglandin E3 (C) for 24 h, followed by assessment of cell viability using the CCK‐8 assay. Cell viability was normalized to the corresponding untreated control, which was set as 100%. Data are presented as mean ± SEM. *n* = 6. *p < 0.05 versus the untreated control group.
**Figure S3:** Transepithelial electrical resistance of porcine intestinal epithelial (IPEC‐1) cells in response to three key metabolites. IPEC‐1 cells were treated with Tyr‐Phe (10 μM), 2‐hydroxyoctadecanoic acid (2‐HA, 8 μM), or prostaglandin E3 (PE3, 5 nM) for 24 h. ΔTEER values were calculated as the relative change from baseline (0 h) to 24 h and normalized to the control group. Data are shown as mean ± SEM. *n* = 3. *p < 0.05 and ***p < 0.001 versus control.

## Data Availability

The 16S rRNA gene sequencing data have been deposited in the NCBI Sequence Read Archive (SRA) under accession numbers PRJNA1338811 and PRJNA1338967. The RNA‐seq data are also available in the NCBI SRA under accession number PRJNA1338998. Additionally, the raw sequencing data have also been archived in ZENODO. The 16S rRNA gene sequencing raw data are available at https://doi.org/10.5281/zenodo.17364259 and https://doi.org/10.5281/zenodo.17364338, and the RNA‐seq data at https://doi.org/10.5281/zenodo.17364736. The metabolomics data have been deposited in MetaboLights under accession number MTBLS13117 (https://www.ebi.ac.uk/metabolights/reviewer3756610c‐c3c5‐4378‐baef‐281043be18c5).
