# Supplementary material for: Transplantation of Elderly Human Gut Microbiota Into Pigs Reprograms Intestinal Barrier Function, Plasma Metabolome, and Gut Mucosal Transcriptomic Landscape
Source: Aging Cell. 2026 Sep 23;25(10):e70725. doi: 10.1111/acel.70725 (PMC13599347; doi:10.1111/acel.70725)
Supplement: Supplementary file 1 [file ACEL-25-e70725-s001.docx]

**Supplementary materials**

**Transplantation of Elderly Human Gut Microbiota into Pigs Reprograms Intestinal Barrier Function, Plasma Metabolome, and Gut Mucosal Transcriptomic Landscape**

Jiaxin Wang ^1,#^, Lingyu Li ^1,#^, Lihua Mei ^1^, Zhining Tang ^1^, Baorui Liao ^1^, Qianye Zhao ^1^, Yuzeng Wang ^1^, Qingyao Fu ^1^, Liu Ren ^1^, Zhian Zhai ^1^, Yifei Xu ^1^, Aoyu Yang ^1^, Shuai Duan ^1^, Zhengyuan Zhai ^3^, Yanling Hao ^2^, Yalin Zhou ^4^, Yajun Xu ^4^, Ying Yang ^1^, Zhenlong Wu ^1^, Yun Ji ^1,*^

^1^ *State Key Laboratory of Animal Nutrition and Feeding, China Agricultural University, Beijing, 100193, China*

^2^ *Key Laboratory of Precision Nutrition and Food Quality, Department of Nutrition and Health, China Agricultural University, Beijing 100193, China*

^3^ *College of Food Science and Nutritional Engineering, China Agricultural University, Beijing 100083, China*

^4^ *Department of Nutrition and Food Hygiene, School of Public Health, Peking University, Beijing 100083, China*

^#^ Contributed equally.

* Corresponding author:

Yun Ji, Ph.D.

State Key Laboratory of Animal Nutrition and Feeding,

China Agricultural University,

Beijing, 100193, China

*Email: [jiyun@cau.edu.cn](mailto:jiyun@cau.edu.cn)

Tel/Fax: +86-10-62731752

**Table S1.** Effects of fecal microbiota transplantation from young or elderly donors on the blood routine indicators of pigs.

| Items | Control | | | Young | | | Old | | |
| --- | --- | --- | --- | --- | --- | --- | --- | --- | --- |
|  | Mean ± SEM | Median | Range | Mean ± SEM | Median | Range | Mean ± SEM | Median | Range |
| RBC, 10^12^/L | 5.73 ± 0.41 | 5.94 | 2.78 | 5.66 ± 0.32 | 5.56 | 1.69 | 6.03 ± 0.24 | 6.07 | 1.68 |
| HCT, % | 28.82 ± 1.30 | 28.55 | 7.40 | 27.32 ± 1.88 | 28.70 | 10.50 | 30.63 ± 1.02 | 30.80 | 6.50 |
| HGB, g/L | 81.67 ± 3.65 | 82.50 | 24.00 | 78.67 ± 6.17 | 86.00 | 36.00 | 87.67 ± 3.93 | 87.00 | 24.00 |
| MCV, fL | 51.18 ± 2.83 | 50.70 | 20.10 | 48.27 ± 1.98 | 48.55 | 13.30 | 51.20 ± 2.51 | 52.65 | 15.10 |
| MCH, pg | 14.57 ± 0.10 | 14.85 | 7.10 | 13.95 ± 1.01 | 13.45 | 7.40 | 14.67 ± 0.91 | 15.20 | 5.00 |
| MCHC, g/L | 283.83 ± 5.31 | 283.00 | 29.00 | 287.67 ± 10.94 | 293.50 | 74.00 | 285.67 ± 4.98 | 287.00 | 33.00 |
| RDW-CV, % | 31.08 ± 3.24 | 33.55 | 19.50 | 34.08 ± 3.14 | 37.30 | 18.90 | 32.88 ± 4.13 | 33.20 | 22.70 |
| NRBC%, % | 1.12 ± 0.28 | 1.40 | 1.70 | 0.62 ± 0.14 | 0.7 | 0.80 | 0.57 ± 0.20 | 0.35 | 1.20 |
| NRBC, 10^9^/L | 0.24 ± 0.07 | 0.27 | 0.41 | 0.10 ± 0.02 | 0.11 | 0.11 | 0.16 ± 0.09 | 0.08 | 0.57 |
| WBC, 10^9^/L | 21.14 ± 1.71 | 20.14 | 11.70 | 16.75 ± 1.47 | 16.65 | 7.76 | 25.41 ± 5.12 | 24.59 | 33.39 |
| NEUT%, % | 43.03 ± 3.48 | 41.90 | 23.30 | 33.80 ± 5.74 | 37.55 | 38.10 | 46.38 ± 6.46 | 45.90 | 40.30 |
| LYMPH%, % | 43.33 ± 3.14 | 44.15 | 19.70 | 49.57 ± 2.72 | 49.05 | 19.30 | 44.22 ± 5.91 | 43.30 | 39.60 |
| MONO%, % | 6.68 ± 0.97 | 6.85 | 6.50 | 6.05 ± 0.79 | 6.25 | 4.40 | 5.88 ± 0.40 | 5.70 | 2.80 |
| EO%, % | 6.47 ± 2.58 | 3.70 | 16.70 | 10.20 ± 8.14 | 2.45 | 50.40 | 3.03 ± 0.93 | 2.50 | 6.20 |
| BASO%, % | 0.48 ± 0.09 | 0.43 | 0.60 | 0.38 ± 0.06 | 0.43 | 0.30 | 0.48 ± 0.03 | 0.48 | 0.20 |
| NEUT, 10^9^/L | 9.05 ± 0.9^ab^ | 8.64 | 5.77 | 5.51 ± 1.08^a^ | 5.52 | 7.33 | 13.30 ± 3.97^b^ | 11.19 | 25.18 |
| LYMPH, 10^9^/L | 9.22 ± 1.14 | 8.88 | 7.83 | 8.16 ± 0.52 | 7.81 | 3.18 | 9.82 ± 1.00 | 9.59 | 6.34 |
| MONO, 10^9^/L | 1.46 ± 0.32 | 1.35 | 2.23 | 1.03 ± 0.19 | 0.87 | 1.13 | 1.45 ± 0.26 | 1.43 | 1.89 |
| EO, 10^9^/L | 1.31 ± 0.51 | 0.84 | 3.42 | 1.98 ± 1.68 | 0.36 | 10.31 | 0.72 ± 0.28 | 0.60 | 1.83 |
| BASO, 10^9^/L | 0.10 ± 0.02^ab^ | 0.10 | 0.11 | 0.07 ± 0.01^a^ | 0.06 | 0.08 | 0.12 ± 0.02^b^ | 0.12 | 0.18 |
| PLT, K/μL | 1652.33 ± 694.78 | 803.50 | 4291.00 | 1488.00 ± 867.87 | 605.00 | 5275.00 | 1371.00 ± 498.42 | 821.50 | 3229.00 |
| MPV, fL | 11.55 ± 1.16 | 10.90 | 6.70 | 9.48 ± 1.13 | 8.45 | 7.10 | 11.52 ± 1.37 | 10.55 | 7.40 |
| PCT, % | 2.25 ± 1.13 | 0.76 | 6.95 | 1.90 ± 1.38 | 0.52 | 8.38 | 1.83 ± 0.84 | 0.81 | 5.30 |
| PLT-I, 10^9^/L | 1652.33 ± 694.78 | 803.50 | 4291.00 | 1488.00 ± 867.87 | 605.00 | 5275.00 | 1371.00 ± 498.42 | 821.50 | 3229.00 |
| P-LCR, % | 41.28 ± 10.01 | 34.90 | 57.20 | 22.92 ± 10.34 | 13.90 | 67.40 | 40.17 ± 11.87 | 31.70 | 63.30 |

Note： RBC, Red blood cell; HCT, Hematocrit; HGB, Hemoglobin; MCV, Mean corpuscular volume; MCH, Mean corpuscular hemoglobin; MCHC, Mean corpuscular hemoglobin concentration; RDW-CV, Red cell distribution width-coefficient of variation; RDW-SD, Red cell distribution width-standard deviation; NRBC%, Nucleated red blood cell percentage; NRBC, Nucleated red blood cell count; WBC, White blood cell count; NEUT%, Neutrophil percentage; LYMPH%, Lymphocyte percentage; MONO%, Monocyte percentage; EO%, Eosinophil percentage; BASO%, Basophil percentage; NEUT, Neutrophil count; LYMPH, Lymphocyte count; MONO, Monocyte count; EO, Eosinophil count; BASO, Basophil count; PLT, Platelet count; MPV, Mean platelet volume; PCT, Plateletcrit; PLT-I, Platelet index; P-LCR, Platelet large cell ratio. n = 6. Different superscript letters within the same row indicate statistically significant differences (*P <* 0.05), while data without superscript letters in the same row show no significant difference (*P* > 0.05).

**Table S2.** Impacts of human fecal microbiota transplantation on blood biochemical parameters in pigs.

| Items | Control | | | Young | | | Old | | |
| --- | --- | --- | --- | --- | --- | --- | --- | --- | --- |
|  | Mean ± SEM | Median | Range | Mean ± SEM | Median | Range | Mean ± SEM | Median | Range |
| ALT, U/L | 49.15 ± 5.37 | 48.90 | 36.00 | 55.73 ± 6.29 | 56.05 | 45.70 | 55.52 ± 2.89 | 56.70 | 18.30 |
| AST, U/L | 50.42 ± 6.02 | 46.80 | 39.90 | 62.13 ± 6.37 | 57.25 | 36.90 | 51.80 ± 3.70 | 51.55 | 23.00 |
| TP, g/dL | 5.52 ± 0.18 | 5.63 | 1.30 | 5.15 ± 0.16 | 5.22 | 0.96 | 5.43 ± 0.16 | 5.37 | 0.87 |
| ALB, g/dL | 3.72 ± 0.17 | 3.82 | 1.14 | 3.57 ± 0.13 | 3.51 | 0.75 | 3.84 ± 0.16 | 3.82 | 1.17 |
| GLB, g/dL | 1.80 ± 0.19 | 1.62 | 1.23 | 1.58 ± 0.13 | 1.53 | 0.84 | 1.59 ± 0.10 | 1.49 | 0.63 |
| A/G | 2.19 ± 0.25 | 2.26 | 1.64 | 2.34 ± 0.23 | 2.29 | 1.60 | 2.48 ± 0.21 | 2.51 | 1.31 |
| GGT, U/L | 84.21 ± 36.03 | 48.19 | 229.52 | 50.92 ± 2.96 | 54.00 | 19.29 | 71.28 ± 22.35 | 46.21 | 143.33 |
| CK, U/L | 638.73 ± 144.71 | 571.30 | 866.30 | 422.02 ± 54.36 | 421.20 | 416.40 | 393.73 ± 37.95 | 377.15 | 263.80 |
| GLU, mg/dL | 87.18 ± 2.43 | 84.38 | 15.09 | 87.20 ± 2.94 | 87.68 | 18.32 | 86.57 ± 4.48 | 83.13 | 26.12 |
| UREA, mg/dL | 5.64 ± 0.87 | 5.32 | 5.38 | 7.48 ± 1.13 | 7.26 | 7.69 | 7.51 ± 1.53 | 6.73 | 9.65 |
| CREA, mg/dL | 0.34 ± 0.01 | 0.35 | 0.08 | 0.34 ± 0.03 | 0.34 | 0.21 | 0.34 ± 0.02 | 0.35 | 0.13 |
| CHOL, mg/dL | 60.68 ± 3.27 | 59.40 | 19.05 | 59.23 ± 3.30 | 57.95 | 21.80 | 57.87 ± 2.36 | 57.40 | 14.54 |
| TG, mg/dL | 29.20 ± 2.36 | 28.40 | 15.90 | 31.00 ± 5.27 | 27.80 | 30.90 | 31.17 ± 2.83 | 32.10 | 17.70 |
| HDL-C, mg/dL | 29.68 ± 0.93 | 29.83 | 5.85 | 28.95 ± 2.32 | 29.89 | 14.39 | 26.60 ± 1.42 | 26.46 | 10.68 |
| LDL-C, mg/dL | 39.92 ± 3.36 | 37.84 | 19.97 | 39.59 ± 1.92 | 38.67 | 13.47 | 40.00 ± 3.12 | 36.57 | 19.99 |
| TBA, μmol/L | 19.05 ± 4.60 | 17.05 | 29.80 | 16.08 ± 4.56 | 17.15 | 30.20 | 23.40 ± 5.66 | 25.00 | 29.90 |
| FRU, μmol/L | 209.69 ± 5.98 | 210.33 | 42.25 | 201.62 ± 4.63 | 204.33 | 27.32 | 215.46 ± 2.95 | 216.52 | 21.13 |
| NEFA, mmol/L | 0.17 ± 0.04 | 0.13 | 0.24 | 0.18 ± 0.01 | 0.18 | 0.09 | 0.22 ± 0.05 | 0.18 | 0.32 |

Note: ALT, Alanine aminotransferase; AST, Aspartate aminotransferase; TP, Total protein; ALB, Albumin; GLB, Globulin; A/G, Albumin-to-globulin ratio; GGT, Gamma-glutamyl transferase; CK, Creatine kinase; GLU, Glucose; UREA, Urea; CREA, Creatinine; CHOL, Cholesterol; TG, Triglycerides; HDL-C, High-density lipoprotein cholesterol; LDL-C, Low-density lipoprotein cholesterol; TBA, Total bile acids; FRU, Fructosamine; NEFA, Non-esterified fatty acids. n = 6. Different superscript letters within the same row indicate statistically significant differences (*P <* 0.05), while data without superscript letters in the same row show no significant difference (*P* > 0.05).

**Table S3.** Fold change of the 7 hub genes identified.

| Gene Name | Gene Description | FC(Old/Young) | Log_2_FC(Old/Young) | *P* value |
| --- | --- | --- | --- | --- |
| *MX2* | MX dynamin like GTPase 2 | 0.451 | -1.14742 | 0.019623 |
| *ISG15* | ISG15 ubiquitin like modifier | 0.387 | -1.36793 | 0.013552 |
| *IFI6* | interferon alpha inducible protein 6 | 0.349 | -1.51977 | 0.001852 |
| *IFIT1* | interferon-induced protein with tetratricopeptide repeats 1 | 0.43 | -1.21853 | 0.006996 |
| *OAS1* | 2'-5'-oligoadenylate synthetase 1 | 0.376 | -1.41085 | 0.001997 |
| *DHX58* | DExH-box helicase 58 | 0.464 | -1.10927 | 0.02277 |
| *ISG12(A)* | putative ISG12(a) protein | 0.432 | -1.211 | 0.006189 |

**Figure S1.** ACE-based rarefaction curves of microbial communities following antibiotics treatment and fecal microbiota transplantation. (A) ACE rarefaction curves of control and antibiotics-treated groups. (B) ACE rarefaction curves of recipients after FMT from young or elderly donors. Each curve represents an individual sample. Abx, antibiotics. ACE, Abundance-based Coverage Estimator.

**Figure S2.** Effects of treatment with different concentrations of three key metabolites on the viability of porcine intestinal epithelial (IPEC-1) cells. IPEC-1 cells were treated with serial concentrations of Tyr-Phe (A), 2-hydroxyoctadecanoic acid (B), or prostaglandin E3 (C) for 24 h, followed by assessment of cell viability using the CCK-8 assay. Cell viability was normalized to the corresponding untreated control, which was set as 100%. Data are presented as mean ± SEM. n = 6. **P* < 0.05 versus the untreated control group.

**Figure S3.** Transepithelial electrical resistance of porcine intestinal epithelial (IPEC-1) cells in response to three key metabolites. IPEC-1 cells were treated with Tyr-Phe (10 μM), 2-hydroxyoctadecanoic acid (2-HA, 8 μM), or prostaglandin E3 (PE3, 5 nM) for 24 h. ΔTEER values were calculated as the relative change from baseline (0 h) to 24 h and normalized to the control group. Data are shown as mean ± SEM. n = 3. **P* < 0.05 and ****P* < 0.001 versus control.
